# Differences in Esophageal Cancer Surgery in Terms of Surgical Approach and Extent of Lymphadenectomy: Findings of an International Survey

**DOI:** 10.1245/s10434-019-07316-9

**Published:** 2019-03-21

**Authors:** A. S. van Rijswijk, E. R. C. Hagens, D. L. van der Peet, M. I. van Berge Henegouwen, S. S. Gisbertz

**Affiliations:** 1 Amsterdam UMC, Department of Surgery, Cancer Center Amsterdam, University of Amsterdam, Amsterdam, The Netherlands; 2 Amsterdam UMC, Department of Surgery, Cancer Center Amsterdam, VU University Amsterdam, Amsterdam, The Netherlands

## Abstract

**Introduction:**

Esophagectomy and lymphadenectomy are essential parts of the multimodal treatment of esophageal carcinoma with curative intent. Treatment regimens vary globally and are subject to debate. A global survey was designed to gain insight into current practice.

**Methods:**

Fifty-seven international expert upper gastrointestinal surgeons received a personal invitation to participate in the survey, which focused on demographics and experience; extent of lymphadenectomy in adeno and squamous cell carcinoma; use of classification systems; neoadjuvant therapy; surgical approach; and specimen handling.

**Results:**

The response rate was 88% (50/57 surgeons), with a mean age of 51.6 years and a median number of 15 years of experience in esophageal surgery. The variety in the extent of lymphadenectomy in proximal, middle and distal squamous cell carcinoma, and Siewert I, II and III adenocarcinoma, was considerable. The number of different combinations of lymph node (LN) stations that were resected in the same tumor was high, while the number of surgeons who removed the exact same combination of LN stations was low. Illustrative is Siewert I adenocarcinoma, in which 27 unique combinations of LN stations were resected, with a maximum of two surgeons performing the exact same dissection. Use of neoadjuvant therapy, surgical approach, and specimen handling also show great variety among participants.

**Conclusion:**

There is no uniform, worldwide strategy for surgical treatment of esophageal cancer. The extent of lymphadenectomy shows great variation for both histologic types. An international observational study is needed to provide evidence on the distribution pattern of lymph node metastases in esophageal cancer and the necessary extent of lymphadenectomy.

**Electronic supplementary material:**

The online version of this article (10.1245/s10434-019-07316-9) contains supplementary material, which is available to authorized users.

Esophageal carcinoma is among the world’s most prevalent and fatal malignancies, with 455,800 new cases and 400,200 esophageal cancer-related deaths worldwide in 2012.^1^^–^4 Esophageal cancer is still associated with a poor prognosis, although survival has improved considerably with the introduction of multimodal treatment.^5^ One of the most important prognostic parameters is the presence of lymph node (LN) metastases; 6^–^8 however, there is no consensus on the optimal extent of lymphadenectomy and surgical procedure.^9^ Surgical approach depends on tumor characteristics, patient factors, local or surgeon’s preference, and the desired extent of lymphadenectomy.^10^,^11^ Lymphadenectomy is a pivotal step in the surgical treatment of esophageal cancer as it promotes proper staging and contributes to locoregional tumor control. Although the lymphadenectomy is perceived as an essential step in the treatment of esophageal cancer, the dimension has been subject to worldwide debate. In theory, the extent of lymphadenectomy should be based on the metastatic lymph nodal map. Notwithstanding the efforts of many to elucidate the distribution pattern of LN metastasis in esophageal adenocarcinoma (AC) and squamous cell carcinoma (SCC), evidence on the dissemination route remains incohesive, especially for AC. This is mainly due to the heterogeneity of the available evidence and the use of different LN classifications, which makes data incomparable in meta-analysis.^6^,^12^^–^21 Current practice in leading esophageal cancer centers varies, but to what extent is not well-known. The objective of this study was to provide insight into the international treatment of esophageal cancer, with an emphasis on the surgical approach and the extent of lymphadenectomy.

## Methods

An initial working group identified high-volume international esophageal surgical centers and individual surgeons. These centers and surgeons were contacted in 2014 to participate in the TIGER collaboration with the aim of determining the optimal lymphadenectomy in esophageal cancer patients. The main project of this collaboration is the TIGER study (NCT03222895). It was agreed to participate in an international survey to get insight into current clinical practice. In January 2015, all participants of this focus group were invited by e-mail to participate in the electronic survey and received a personal link to fill out the survey. The survey focused on demographics and experience in general, as well as esophageal surgery; the extent of lymphadenectomy for proximal, middle and distal SCC, and Siewert I, II and III AC; the application of neoadjuvant therapy; the surgical approach per primary tumor location and histologic tumor type; and the resected specimen handling. Questions on LN classification systems, the extent of lymphadenectomy, and the use of neoadjuvant treatment were mandatory. The majority of the questions were multiple choice. In the demographical section, questions were answered by providing numbers or percentages. In the section on lymphadenectomy, a dropdown of all LN stations according the 9th edition of the Japanese Society of Esophageal Diseases (JSED; at present the Japanese Esophageal Society), or the American Joint Committee on Cancer (AJCC) 6th edition was offered and respondents were asked to mark all the LN stations that are resected in distal, middle and proximal SCC, and in Siewert I, II and III AC. All users of the 6th edition of the AJCC and 9th edition of the JSED were offered their own classification system to answer these questions. If a respondent used a different classification system other than the AJCC 6th edition or the JSED 9th edition, the respondent was redirected to use the JSED 9th edition to complete questions on the dimension of the lymphadenectomy. In the section on surgical approach, a dropdown menu was designed to combine the surgical approach and type and location of anastomosis. The content of the questionnaire is shown in electronic supplementary material 2.

The questionnaire was designed and distributed using SoGoSurvey, a web-based program to design surveys. All data were gathered anonymously and collected in an Excel file (version 2016; Microsoft Corporation, Redmond, WA, USA) and then converted to an SPSS file (version 23; IBM Corporation, Armonk, NY, USA). The first invitation was sent on 30 January 2015, and reminders were sent after 2 weeks and 3 and 6 months. If the survey was not completed by 30 September 2015, a recipient was considered to be a non-responder.

### Statistical Analysis

All data were handled anonymously. Data analysis was performed using SPSS statistical software. For descriptive statistics, the mean and standard deviation (SD) was used for normally distributed data, and the median and interquartile range (IQR) was used for skewed data. Data were also analyzed separately for continent of origin, case volume, and experience of the respondents.

## Results

### Demographics

**Fig. 1:**
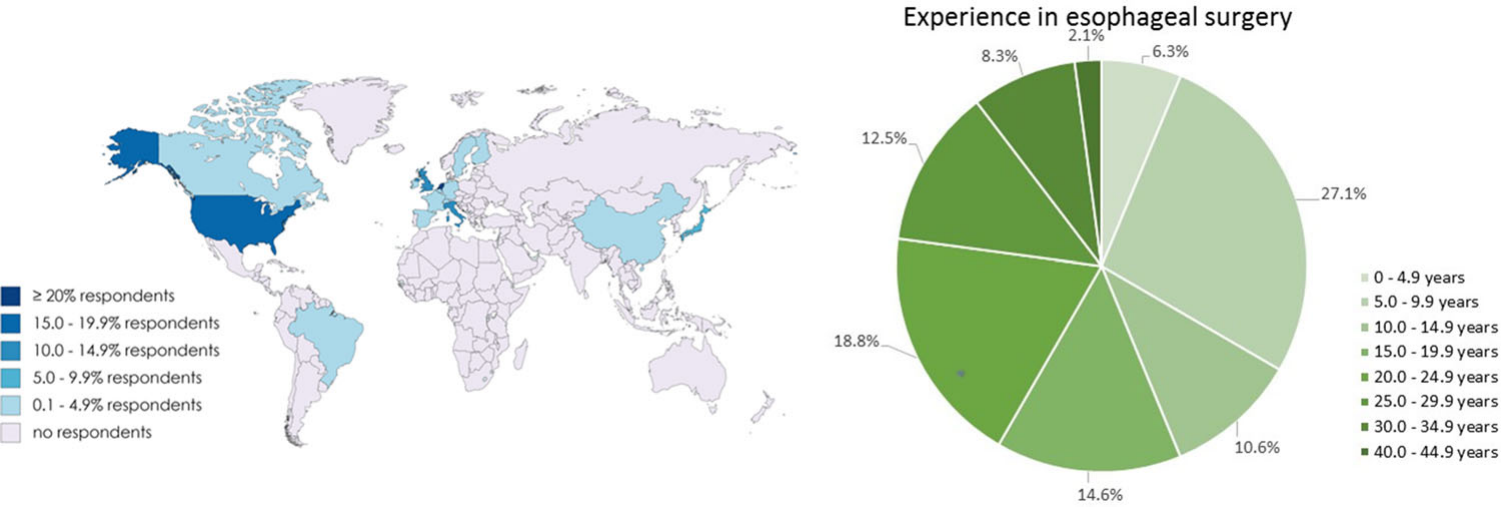
Experience of participants in esophageal surgery, in years, and their worldwide distribution. Map created with MapChart (https://mapchart.net)

The response rate was 87.7%, with 50 of 57 surgeons responding from Europe (68.0%), North America (18.0%), South America (8.0%), and Asia (10.0%) (Fig. 1). The seven non-respondents came from Asia (5) and Europe (2). Mean age was 51.6 years (SD ± 7.7). Forty-one surgeons worked in a tertiary referral center (82.0%), eight in a secondary referral center (16.0%), and one in a local hospital (2.0%). Mean experience in general surgery and esophageal surgery was 18.1 (SD ± 10.8) and 16.2 (SD ± 9.2) years, respectively (Fig. 1). In their medical centers, a median of 55.0 (IQR 33.5–90.0) esophagectomies were performed annually. The number of esophageal cancer surgeons per medical center ranged from one to eight. Thirty-five surgeons had a personal annual case volume of 30 or higher.

### Surgical Techniques

All respondents administered neoadjuvant therapy. Substantial variation was seen in the neoadjuvant regimens administered for the different types of esophageal cancer (Table 1). The CROSS regimen was most often used for patients with Siewert I AC, middle-esophageal SCC, and distal SCC among European and North American respondents. The MAGIC scheme was more often used in cases of Siewert III AC, specifically for Europe (64.7% of European respondents), North America (71.4%), and South America (all respondents). None of the Asian respondents indicated they use the MAGIC scheme for any type of tumor. A minimally invasive approach was preferred by 32 (62.0%) participants. Within Europe, 70.6% of participants performed minimally invasive techniques, 33.3% within North America, one of two respondents in South America, and 60.0% of respondents from Asia. Open esophagectomy was performed by 13 (26.0%) surgeons. Among the European respondents, 14.7% performed an open esophagectomy, 66.7% of the North American respondents, one of two participants from South America, and 20% of Asian respondents. Six (12%) surgeons did not answer the question—five from Europe and one from Asia. The minimally invasive transthoracic approach with intrathoracic anastomosis was performed most in Siewert I and II AC, while the transthoracic approach with cervical anastomosis was performed most in proximal and middle SCC (Table 1).

**Table 1:** Regimens of neoadjuvant treatment administered and surgical approach preferred by responding surgeons in the different types of esophageal cancer

	CROSS	MAGIC	Other	Missing	Total	Total gastrectomy	Transhiatal	Transthoracic, intrathoracic anastomosis	Transthoracic, cervical anastomosis	Definitive chemoradiation	Other
Proximal SCC	29 (58)	0 (0)	20 (40)	1 (2)	50 (100)	0	1 (2)	2 (4)	35 (70)	7 (14)	5 (10)
Middle SCC	34 (68)	0 (0)	16 (32)	2 (4)	50 (100)	0	0	11 (22)	35 (70)	0	4 (8)
Distal SCC	34 (68)	0 (0)	12 (30)	1 (2)	50 (100)	0	2 (4)	31 (32)	15 (30)	0	4 (8)
SW 1	29 (68)	6 (12)	14 (28)	1 (2)	50 (100)	0	2 (4)	34 (68)	14 (28)	0	0
SW 2	22 (44)	11 (22)	16 (32)	1 (2)	50 (100)	1 (2)	7 (14)	30 (60)	4 (8)	0	7 (14)
SW 3	6 (12)	30 (60)	12 (28)	2 (4)	50 (100)	31 (62)	3 (6)	6 (12)	0	0	7 (14)

Data are expressed as *n* (%)

*AC* adenocarcinoma, *SCC* squamous cell carcinoma, *SW* Siewert

### Lymphadenectomy Per Lymph Node Station

**Fig. 2:**
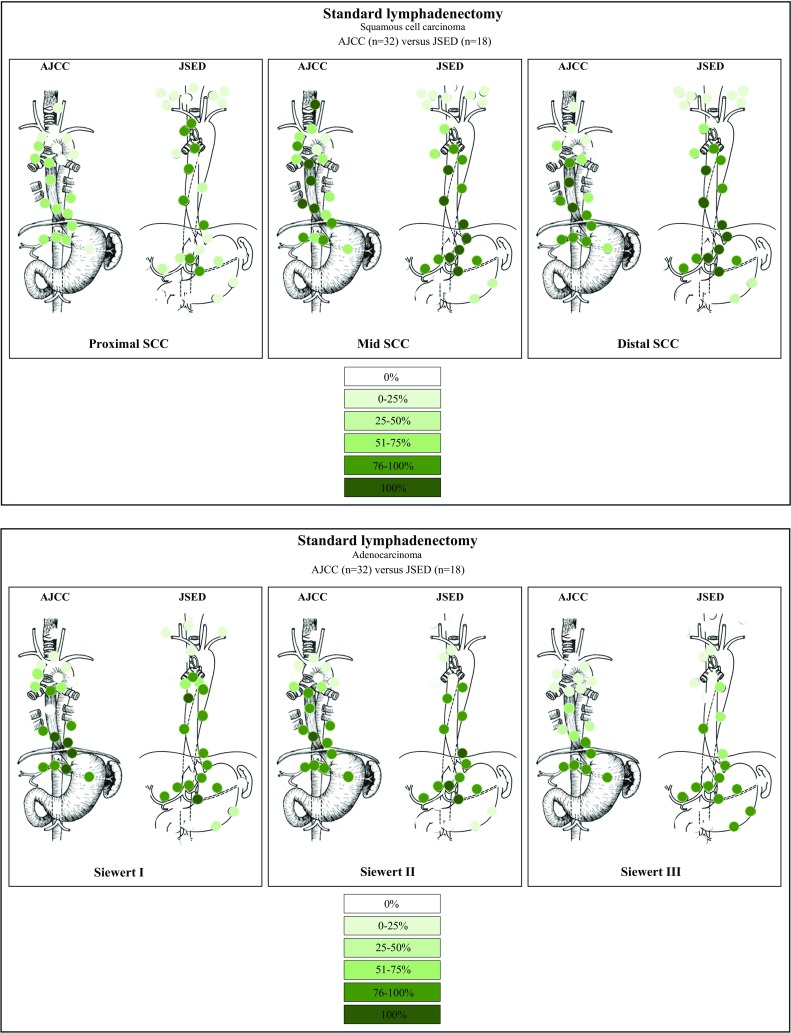
Extent of a standard lymphadenectomy in proximal, middle and distal squamous cell carcinoma, and in Siewert I, II and III adenocarcinoma reported for AJCC 6th edition (*n* = 32) and JSED 9th edition (*n* = 18) users separately. *AJCC* American Joint Committee on Cancer, *JSED* Japanese Society of Esophageal Diseases, *SCC* squamous cell carcinoma

Thirty-two surgeons used the AJCC 6th edition to classify LNs, 14 surgeons used the JSED 9th edition, 1 surgeon used a combination of both, and 3 surgeons used the AJCC 7th edition. Notably, the latter four were redirected to the JSED 9th edition to report their standard lymphadenectomy since this classification system is more comprehensive. Standard lymphadenectomy is depicted in Tables 2 and 3, and visualized in Fig. 2. Within the group of AJCC 6th edition users, over 75% of respondents resected stations 7, 8M and 8L in cases of proximal SCC. All AJCC 6th edition users resected stations 7, 8M, 8L and 9 in the treatment of a middle-esophageal SCC. Stations 8M and 8L were part of the standard lymphadenectomy in distal SCC in all AJCC 6th edition users. In Siewert I AC, all respondents resected stations 8M, 8L, 15, 16 and 17, over 90% resected these same stations in cases of Siewert II AC, and over 90% resected stations 15–20 in Siewert III AC.

**Table 2:** Overview of lymph node stations resected by surgeons using the AJCC 6th edition in a standard lymphadenectomy

	Proximal	Middle	Distal	SW1	SW2	SW3
1 Supraclavicular nodes	3 (9.4)	0	0	0	0	0
2R Right upper paratracheal nodes	8 (25.0)	24 (75.0)	12 (37.5)	7 (21.9)	3 (9.4)	0
2L Left upper paratracheal nodes	18 (56.2)	15 (46.9)	7 (21.9)	2 (6.2)	1 (3.1)	0
3P Posterior mediastinal nodes	19 (59.4)	21 (65.6)	14 (43.8)	12 (37.5)	6 (18.8)	0
4R Right lower paratracheal nodes	23 (71.9)	28 (87.5)	23 (71.9)	20 (62.5)	15 (46.9)	3 (9.4)
4L Left lower paratracheal nodes	19 (59.4)	22 (68.8)	19 (59.4)	15 (46.9)	10 (31.3)	3 (9.4)
5 Aortopulmonary nodes	14 (43.8)	15 (46.9)	14 (43.8)	11 (34.4)	6 (18.8)	1 (3.1)
6 Anterior mediastinal nodes	7 (21.9)	7 (21.9)	7 (21.9)	6 (18.8)	4 (12.5)	2 (6.2)
7 Subcarinal nodes	24 (75.0)	32 (100)	31 (96.9)	31 (96.9)	27 (84.4)	8 (25)
8 M Middle paraesophageal nodes	24 (75.0)	32 (100)	32 (100)	32 (100)	30 (93.8)	12 (37.5)
8L Lower paraesophageal nodes	24 (75.0)	32 (100)	32 (100)	32 (100)	31 (96.9)	21 (65.6)
9 Pulmonary ligament nodes	22 (68.8)	32 (100)	29 (90.6)	29 (90.6)	27 (84.4)	15 (46.9)
10R Right tracheobronchial nodes	17 (53.1)	28 (87.5)	22 (68.7)	22 (68.7)	19 (59.4)	7 (21.9)
10L Left tracheobronchial nodes	14 (43.8)	23 (71.9)	18 (56.2)	20 (62.5)	17 (53.1)	6 (18.8)
15 Diaphragmatic nodes	23 (71.9)	21 (65.6)	31 (96.9)	32 (100)	31 (96.9)	29 (90.6)
16 Paracardial nodes	22 (68.8)	31 (96.9)	31 (96.9)	32 (100)	31 (96.9)	29 (90.6)
17 Left gastric nodes	23 (71.9)	30 (93.8)	31 (96.9)	32 (100)	31 (96.9)	31 (96.9)
18 Common hepatic nodes	19 (59.4)	31 (96.9)	27 (84.4)	29 (90.6)	28 (87.5)	30 (93.8)
19 Splenic nodes	14 (43.8)	24 (75.0)	22 (68.8)	25 (78.1)	25 (78.1)	29 (90.6)
20 Celiac nodes	18 (56.2)	19 (59.4)	28 (87.5)	30 (93.8)	28 (87.5)	31 (96.9)

Data are expressed as *n* (%)

*AJCC* American Joint Committee on Cancer, *L* left, *M* middle, *P* Posterior, *R* right, *SW* Siewert

**Table 3:** Overview of lymph node stations resected by surgeons using the JSED 9th edition in a standard lymphadenectomy

	Proximal	Middle	Distal	SW1	SW2	SW3
100 Superficial cervical (R/L)	9 (50.0)	2 (11.1)	1 (5.6)	0	0	0
101 Cervical paraesophageal (R/L)	13 (72.2)	6 (33.3)	2 (11.1)	2 (11.1)	0	0
102 Deep cervical (R/L)	9 (50.0)	3 (16.7)	1 (5.6)	0	0	0
103 Peripharyngeal (R/L)	5 (27.8)	0	0	0	0	0
104 Supraclavicular	9 (50.0)	4 (22.2)	2 (11.1)	1 (5.6)	0	0
105 Upper thoracic paraesophageal	15 (83.3)	17 (94.4)	12 (66.7)	7 (38.9)	4 (22.2)	2 (11.1)
108 Middle thoracic paraesophageal	15 (83.3)	18 (100)	18 (100)	18 (100)	16 (88.9)	5 (27.8)
110 Lower thoracic paraesophageal	15 (83.3)	18 (100)	18 (100)	17 (94.4)	18 (100)	15 (83.3)
106 Recurrent nerve (R)	16 (88.9)	15 (83.3)	10 (55.6)	5 (27.8)	0	0
106 Recurrent nerve (L)	13 (72.2)	14 (77.8)	8 (44.4)	4 (22.2)	0	1 (5.6)
106 Pretracheal	8 (44.4)	7 (38.9)	3 (16.7)	3 (16.7)	1 (5.6)	1 (5.6)
106 Tracheobronchial (L)	13 (72.2)	12 (66.7)	12 (66.7)	9 (50.0)	4 (22.2)	1 (5.6)
107 Bifurcational	15 (83.3)	18 (100)	17 (94.4)	17 (94.4)	12 (66.7)	5 (27.8)
109 Main stem bronchus (R/L)	10 (55.6)	13 (72.2)	10 (55.6)	10 (55.6)	5 (27.8)	1 (5.6)
111 Supradiaphragmatic	14 (77.8)	17 (94.4)	18 (100)	17 (94.4)	18 (100)	13 (72.2)
112 Posterior mediastinal	12 (66.7)	16 (88.9)	17 (94.4)	17 (94.4)	16 (88.9)	13 (72.2)
113 Ligamentum arteriosum	4 (22.2)	6 (33.3)	3 (16.7)	5 (27.8)	3 (16.7)	1 (5.6)
114 Anterior mediastinal	2 (11.1)	3 (16.7)	3 (16.7)	2 (11.1)	1 (5.6)	1 (5.6)
1.2 Cardiac (R/L)	5 (27.8)	17 (94.4)	18 (100)	17 (94.4)	17 (94.4)	16 (88.9)
3 Lesser curvature	14 (77.8)	18 (100)	18 (100)	18 (100)	18 (100)	17 (94.4)
4 Greater curvature	4 (22.2)	6 (33.3)	7 (38.9)	8 (44.4)	9 (50.0)	15 (83.3)
7 Left gastric artery	14 (77.8)	18 (100)	18 (100)	17 (94.4)	18 (100)	17 (94.4)
8 Common hepatic artery	5 (27.8)	15 (83.3)	16 (88.9)	16 (88.9)	16 (88.9)	17 (94.4)
11 Splenic artery	5 (27.8)	13 (72.2)	15 (83.3)	16 (88.9)	16 (88.9)	16 (88.9)
9 Celiac artery	8 (44.4)	15 (83.3)	16 (88.9)	17 (94.4)	17 (94.4)	17 (94.4)

Data are expressed as *n* (%)

*JSED* Japanese Society of Esophageal Diseases, *L* left, *R* right, *SW* Siewert

Over 80% of users of the JSED 9th edition indicated they resected stations 105, 108, 110, 106R and 107 in cases of proximal SCC; all respondents resected stations 108, 110, 107, 3 and 7 in middle-esophageal SCC. In a distal tumor, stations 108, 110, 111, 3 and 7 were part of a standard nodal dissection in all JSED users. In Siewert I AC, all respondents resected stations 108, 3 and 7. Stations 110, 111, 3 and 7 were resected in Siewert II AC and over 90% of the JSED 9th users resected stations 3, 7, 8 and 9 in cases of Siewert III AC.

### Possible Combinations of Lymph Node Stations

Within the group of AJCC 6th edition users, the highest number of surgeons who performed the exact same lymphadenectomy was seven (reported for Siewert III AC), of which the mean resected number of LN stations was 8.8 (SD ± 3.6). For JSED 9th edition users, the highest number of surgeons who performed the exact same lymphadenectomy was six (reported for Siewert III AC), and the mean number of resected LN stations was 9.8 (SD ± 3.1) (Table 4).

**Table 4:** Variety seen in lymphadenectomies for the different types of SCC and AC, according to use of the AJCC 6th edition [*n* = 32] or JSED 9th edition [*n* = 14]

		Number of combinations of resected LN stations	Mean number of resected LN stations (SD)	Number of surgeons who do not resect any LNs	Highest number of surgeons with the same combination of resected nodes
AJCC proximal SCC	[*n* = 32]	21	14.2 (4.5)	8	3
JSED proximal SCC	[*n* = 14]	14	14.9 (5.8)	1	1
AJCC middle SCC	[*n* = 32]	28	13.8 (3.2)	0	3
JSED middle SCC	[*n* = 14]	12	16.4 (2.2)	0	2
AJCC distal SCC	[*n* = 32]	28	13.3 (3.5)	0	3
JSED distal SCC	[*n* = 14]	13	15.2 (2.2)	0	2
AJCC Siewert I	[*n* = 32]	27	13.1 (3.1)	0	2
JSED Siewert I	[*n* = 14]	13	13.8 (2.6)	0	2
AJCC Siewert II	[*n* = 32]	27	11.6 (3.8)	1	3
JSED Siewert II	[*n* = 14]	10	11.3 (1.9)	0	3
AJCC Siewert III	[*n* = 32]	18	8.8 (3.6)	1	7
JSED Siewert III	[*n* = 14]	8	9.8 (3.1)	0	6

*AC* adenocarcinoma, *AJCC* American Joint Committee on Cancer, *JSED* Japanese Society of Esophageal Diseases, *LN* lymph node, *SCC* squamous cell carcinoma, *SD* standard deviation

The highest number of possible combinations of resected LN stations for AJCC 6th edition users was 28, in middle and distal SCC, whereas the lowest number of possible combinations (i.e. the most uniformity) was 18, seen in Siewert III AC. For users of the JSED 9th edition, the highest number of possible combinations was 14, seen in proximal SCC (1 surgeon who did not perform a resection and 13 surgeons who performed a different lymphadenectomy). The lowest number of combinations among JSED 9th edition users was seen in Siewert III AC, with eight possible combinations (Table 4).

### Outcomes Per Origin, Experience, and Case Volume of Respondents

Respondents from Europe and North America indicated their preference for the AJCC classification (79.4% and 88.9% of respondents, respectively). All South American and Asian responders indicated their preference to use the JSED. No major differences between continents were seen in the number of stations surgeons tended to resect. Except for North America, where 66.7% of responders preferred open esophagectomy, respondents from Europe, South America and Asia preferred minimally invasive esophagectomy in ≥ 50% of cases (Table A, electronic supplementary material 1).

When looking at the number of years’ experience, more than half of the respondents in each quartile preferred the AJCC classification and minimally invasive esophagectomies. No difference was seen in the extent of the lymphadenectomy between the different quartiles (Table B, electronic supplementary material 1).

Case volume did not seem to influence the preference of classification; in each quartile, the majority preferred the AJCC. No difference was seen in the extent of the lymphadenectomy and preference in the operation technique between the different quartiles (Table C, electronic supplementary material 1).

## Discussion

This study describes the current practice in the treatment of esophageal cancer, based on the participation of 50 experts in the field of esophageal cancer surgery in an international survey. The data provided by these specialists exposes substantial global differences in the adoption of the various modalities, of which the curative treatment of esophageal cancer is composed. Although all respondents administered neoadjuvant therapy, considerable variety was seen in neoadjuvant regimens for the same tumors (histology and location). Two-thirds of surgeons practice minimally invasive surgery; however, the surgical procedures differ, and the variety in the extent of lymphadenectomy was substantial. The outcome of this study does not only expose the differences seen in the oncologic treatment of esophageal cancer but also reinforces the magnitude of the differences seen. There is apparently a relative lack of information to proceed towards uniform standing and practice in the treatment of esophageal malignancies.

The differences in neoadjuvant therapy can be explained by the trials that have been performed in different countries. In Japan, neoadjuvant chemotherapy with 5-fluorouracil and cisplatin is standard treatment, as reported after publication of the JCOG9204 trial.^22^ In addition, patients in the United Kingdom (UK) are treated with chemotherapy. The MAGIC trial, which compares perioperative chemotherapy and surgery with surgery alone, was performed in the UK; in both the UK and some North American centers, patients are treated with a perioperative chemotherapy scheme based on this trial.^23^ On the contrary, patients from the Netherlands and other European mainland countries are treated with chemoradiotherapy. Since the Dutch CROSS trial, comparing neoadjuvant chemoradiation and surgery with surgery alone, chemoradiotherapy has been implemented as the standard neoadjuvant treatment regimen in these countries.^5^

The differences in surgical approach can be partially explained by the differences in tumor demographics. In Asia and South America, more SCCs are found, which are usually located in the proximal, middle, or distal esophagus, whereas AC, more frequently observed in Europe and North America, is located in the distal esophagus or at the gastroesophageal junction (GEJ).^24^ More proximally located tumors necessitate a transthoracic approach with cervical anastomosis and a more extended lymphadenectomy in the proximal field. There is no evidence that a transthoracic approach is preferred over a transhiatal approach for distal esophageal or GEJ tumors, although a trend towards a better 5-year overall survival can be observed for a transthoracic procedure.^25^,^26^ Both procedures are still being performed, although the majority of the participating surgeons in this study performed a transthoracic resection, with the preferred anastomotic site being in thorax.

In addition, minimally invasive esophagectomy was not equally distributed over the continents. A minimally invasive esophagectomy seems to be preferably performed by European surgeons. This can be explained by the relative overrepresentation of Dutch surgeons in this survey. In the Netherlands, a small and densely populated country, a rapid implementation of minimally invasive esophagectomy has taken place. The percentage of minimally invasive esophagectomies has increased from 31% in 2011 to 98% in 2017 due to good proctoring programs and infrastructure.^27^

When it comes to differences in the extent of lymphadenectomy, a few explanations can be found. First, an important difficulty is encountered in the analysis of data regarding lymphadenectomy. It is challenging to compare standard lymphadenectomies of users of both classification systems as there is no resemblance between the vast number of LN stations of these classifications. In addition, there is no validated tool to translate results from one classification to the other. Ironically, as one of the main aspects of this work is to highlight this problem, the report regarding the data from the present study also suffers from the incomparability of the classification systems used. The JSED 9th edition classification system offers a more detailed map of the LN stations than the 6th edition of the AJCC, especially in the upper mediastinum and cervical areas. The historic perspective of these classifications might elucidate the extent of LNs represented; the JSED was designed as a true esophageal LN map, while the AJCC was based on the nodal map for lung cancer. The Japanese society has constituted a true gastric carcinoma classification system also, which corresponds with the abdominal LNs depicted in the JSED classification for esophageal cancer, and may account for the slightly more detailed abdominal map compared with the abdominal section of the AJCC. Second, and in line with the global discussion on lymphadenectomy, users of the JSED 9th edition (used by 100% of Asian respondents) performed a more extended cervical lymphadenectomy, i.e. a three-field lymphadenectomy as opposed to users of the AJCC 6th or 7th editions (used by 79% of European respondents). This can be attributed to the high incidence of SCC in Asia, and AC in the West, respectively. The differences in the extent of the (cervical) lymphadenectomy, a reflection of Western and Eastern differences, are reported in the comprehensive review by Nafteux et al. on the surgical approach and the optimal extent of the nodal dissection.^28^ Although many noteworthy papers are summarized in this review, the paper by Nafteux et al. illustrates the relative lack of comparable data on this topic and gives insight into the difficulties encountered in evidence-based decision making on the proper extent of lymphadenectomy. The paucity of comparable high-end data on this topic is remarkable as lymphadenectomy has been recognized as a pivotal element of the surgical treatment of esophageal cancer as it constitutes proper staging and local disease control; however, the therapeutic value attributed to lymphadenectomies of different yields remains controversial.^29^^–^31 Although the available evidence illustrates the differences in surgical extent in various parts of the world, data cannot be compared in meta-analysis due to the limitations as mentioned above. However, homogeneous use of classification systems is warranted as it contributes to the comparability of data and a more uniform and evidence-based standing on this subject. Consequently, answers to questions on the behavior of lymphatic metastasis and the optimal resection can be distilled out of global pooling of data.

Regardless of the many differences in the preferred extent of lymphadenectomy, some similarities are also seen. Middle and lower paraesophageal LNs are always resected by all surgeons in both middle and distal SCC and Siewert I AC. Subcarinal nodes are frequently resected in middle and distal SCC and Siewert I AC. In addition, alongside the lower paraesophageal LN stations, most surgeons resect paracardial LN stations and LNs along the lesser curvature and left gastric artery. In Siewert III AC, over 95% of surgeons resect the LN stations along the common hepatic artery and celiac artery.

Noteworthy is that pericardial and cardiac nodes in Siewert III cancers are not resected by over 10% of surgeons. These results are surprising, however a recently published randomized controlled trial found similar results.^32^

When reviewing the differences between experience and case volume, a preference for AJCC in all quartiles is observable. This can be explained by the preference for AJCC use over JSED use in the group of European and North American respondents, who are overrepresented in this survey.

A few limitations of this study have to be addressed. First, assembly of the participant group was not at random. Surgeons and centers were chosen by a working group based on their case volume, scientific contribution, and reputation. This was done to establish the TIGER collaboration with the aim of determining the optimal lymphadenectomy in esophageal cancer patients. Collaborating surgeons were invited to participate in the questionnaire. Although surgeons from four continents participated in the survey, there was an overrepresentation from the ‘West’. Therefore, data are indicative of the global differences in the standard treatment of esophageal cancer, but data are too scarce to reach firm conclusions on the details of the treatment in all parts of the world separately. However, as has been reasoned upon before, this study does suggest intracontinental differences. Even in this selection, with overrepresentation of European countries, the differences in treatment and lymphadenectomy are considerable.

In addition, only two classification systems were offered. Consequently, some users had to fill out the survey without the use of their preferred classification system and were forced to use the JSED 9th edition to map their standard lymphadenectomy. This applied to four surgeons in this survey.

Moreover, the use of two classification systems made the comparison and interpretation of the data more difficult, but, at the same time, prevented more respondents (i.e. all AJCC 6th edition or all JSED 9th edition users) from filling out the questionnaire with a classification system other than the one they were accustomed to.

To date, the AJCC 6th edition has been replaced by the 7th and, recently, 8th editions. Although this is an important limitation, it has to be acknowledged that only three surgeons indicated their preference to use the AJCC 7th edition instead of the 6th edition, one surgeon used a combination of the AJCC 6th edition and JSED 9th edition, and the 8th edition was not yet released at the time of this survey. Even though the 6th edition is outdated, it does not essentially differ from its successors regarding the location of lymph node stations, and is therefore not believed to have influenced the results of this study.

Furthermore, the survey is a result of an international focus group on the surgical treatment of esophageal carcinoma, intended to get insight into current practice and to help design an international observatory study on this topic. Upon invitation, the study group consisted of 57 participants, and therefore the number of invited surgeons was quite limited. However, a high response rate of 88% was reached and participants’ extensive experience in esophageal cancer surgery contributed to the quality of the evidence.

## Conclusions

There is no uniform, worldwide strategy for curative treatment of esophageal cancer, as illustrated by the differences seen in the treatment of 50 experts in the field. The neoadjuvant treatment and surgical approach differ and a great variety is seen in the extent of lymphadenectomy. For scientific purposes, acceptance and implementation of one LN classification system is warranted. An international observational study is needed to provide evidence on the distribution pattern of LN metastases in esophageal cancer and the necessary extent of lymphadenectomy. The multinational TIGER study (NCT 03222895) might provide more information on the LN distribution pattern, providing evidence for esophageal cancer surgeons to determine the optimal strategy for lymphadenectomy.

## Electronic supplementary material

Below is the link to the electronic supplementary material.

Supplementary material 1 (DOCX 24 kb)

Supplementary material 2 (DOCX 66 kb)
