# Supplementary material for: Differences in Esophageal Cancer Surgery in Terms of Surgical Approach and Extent of Lymphadenectomy: Findings of an International Survey
Source: Ann Surg Oncol. 2019 Mar 21;26(7):2063–72. doi: 10.1245/s10434-019-07316-9 (PMC6545175; doi:10.1245/s10434-019-07316-9)
Supplement: Supplementary file 1 — Supplementary material 1 (DOCX 24 kb) [file 10434_2019_7316_MOESM1_ESM.docx]

**Supplement 1. Outcomes per origin, experience and case volume of respondents**

|  | **Continent of origin** | | | |
| --- | --- | --- | --- | --- |
|  | Europe | North America | South America | Asia |
|  | n=34 | n=9 | n=2 | n=5 |
| **Classification** *n (%)* |  |  |  |  |
| JSED 9th edition | 6 (18) | 1 (11) | 2 (100) | 5 (100) |
| AJCC* | 27 (79) | 8 (89) | 0 | 0 |
| Combination of both | 1 (3) | 0 | 0 | 0 |
| **Number of stations** *Median (IQR)* |  |  |  |  |
| proximal SCC | 17 (14-18) | 12 (8-14) | 19, 20^ | 15 (14-19) |
| mid SCC | 16 (14-17) | 11 (9-15) | 17, 19^ | 18 (16-19) |
| distal laag | 14 (13-17) | 9 (8-12) | 14, 15^ | 17 (14-19) |
| SW I AC | 14 (12-15) | 10 (8-11) | 12, 13^ | 15 (10-17) |
| SW II AC | 13 (11-14) | 9 (8-11) | 9, 10^ | 8 (8-9) |
| SW III AC | 9 (6-11) | 8 (8-9) | 7, 9^ | 9 (7-9) |
| **Operation** *n (%)* |  |  |  |  |
| Open esophagectomy | 5 (15) | 6 (67) | 1 (50) | 1 (20) |
| MIE | 24 (71) | 3 (33) | 1 (50) | 3 (60) |
| Question not answered | 5 (15) | 0 | 0 | 1 (20) |

*Table a: Preference of classification, extent of lymphadenectomy and operation technique in relation to continent of origin of respondent.*
* 6th or 7th edition, *AC* Adenocarcinoma, *Dist* Distal, *Mid* Middle, *Prox* Proximal, *SCC* Squamous cell carcinoma, *SW* Siewert, *MIE* Minimally invasive esophagectomy

^ Number of stations resected by the two surgeons from South America

|  | **Years of experience as esophageal surgeon** | | | |
| --- | --- | --- | --- | --- |
|  | 1st quartile 1 – 8 years | 2nd quartile  8 – 15 years | 3rd quartile  15 – 23 years | 4th quartile  24 – 40 years |
|  | n=12 | n=12 | n=12 | n=12 |
| **Classification** *n (%)* |  |  |  |  |
| JSED 9th edition | 1 (8) | 5 (42) | 2 (17) | 5 (42) |
| AJCC* | 11 (92) | 7 (58) | 10 (83) | 6 (50) |
| Combination of both | 0 | 0 | 0 | 1 (8) |
| **Number of stations** *Median (IQR)* |  |  |  |  |
| proximal SCC | 18 (14-18) | 18 (14-20) | 14 (10-18) | 17 (14-17) |
| mid SCC | 17 (14-17) | 16 (13-18) | 16 (11-17) | 17 (13-18) |
| distal laag | 15 (11-17) | 14 (13-16) | 13 (10-16) | 14 (13-18) |
| SW I AC | 14 (12-15) | 13 (12-15) | 12 (10-14) | 14 (11-17) |
| SW II AC | 12 (9-14) | 12 (9-13) | 12 (10-15) | 11 (9-12) |
| SW III AC | 8 (6-11) | 9 (7-11) | 9 (6-10) | 9 (7-11) |
| **Operation** *n (%)* |  |  |  |  |
| Open esophagectomy | 2 (17) | 2 (17) | 3 (25) | 4 (33) |
| MIE | 10 (83) | 7 (58) | 8 (67) | 6 (50) |
| Question not answered | 0 | 3 (25) | 1 (8) | 2 (17) |

*Table b: Preference of classification, extent of lymphadenectomy and operation technique in relation to years or experience.*
* 6th or 7th edition, *AC* Adenocarcinoma, *Dist* Distal, *Mid* Middle, *Prox* Proximal, *SCC* Squamous cell carcinoma, *SW* Siewert, *MIE* Minimally invasive esophagectomy. *Two surgeons did not answer the question regarding years of experience and are therefore not included in this table.*

|  | **Annual personal case volume** | | | |
| --- | --- | --- | --- | --- |
|  | 1st quartile 2 – 25 cases | 2nd quartile  25 – 30 cases | 3rd quartile  25 – 45 cases | 4th quartile  50 – 100 cases |
|  | n=12 | n=12 | n=12 | n=12 |
| **Classification** *n (%)* |  |  |  |  |
| JSED 9th edition | 4 (33) | 4 (33) | 2 (17) | 3 (25) |
| AJCC* | 8 (67) | 8 (67) | 9 (75) | 9 (75) |
| Combination of both | 0 | 0 | 1 (8) | 0 |
| **Number of stations** *Median (IQR)* |  |  |  |  |
| proximal SCC | 15 (13-19) | 16 (12-18) | 16 (15-16) | 17 (14-19) |
| mid SCC | 14 (11-17) | 17 (13-18) | 15 (12-18) | 17 (16-18) |
| distal laag | 13 (11-16) | 15 (11-17) | 14 (11-16) | 15 (13-18) |
| SW I AC | 14 (11-16) | 13 (10-16) | 13 (11-15) | 14 (11-17) |
| SW II AC | 10 (8-13) | 10 (8-13) | 13 (10-13) | 12 (10-15) |
| SW III AC | 7 (6-9) | 9 (7-11) | 9 (7-10) | 8 (6-11) |
| **Operation** *n (%)* |  |  |  |  |
| Open esophagectomy | 2 (17) | 5 (41.5) | 2 (17) | 2 (17) |
| MIE | 9 (75) | 5 (41.5) | 7 (58) | 10 (83) |
| Question not answered | 1 (8) | 2 (17) | 3 (25) | 0 |

*Table c: Preference of classification, extent of lymphadenectomy and operation technique in relation to personal case volume.*
* 6th or 7th edition, *AC* Adenocarcinoma, *Dist* Distal, *Mid* Middle, *Prox* Proximal, *SCC* Squamous cell carcinoma, *SW* Siewert, *MIE* Minimally invasive esophagectomy. *Two surgeons did not answer the question regarding annual personal case volume and are therefore not included in this table.*
