# Supplementary material for: Differences in Esophageal Cancer Surgery in Terms of Surgical Approach and Extent of Lymphadenectomy: Findings of an International Survey
Source: Ann Surg Oncol. 2019 Mar 21;26(7):2063–72. doi: 10.1245/s10434-019-07316-9 (PMC6545175; doi:10.1245/s10434-019-07316-9)
Supplement: Supplementary file 2 — Supplementary material 2 (DOCX 66 kb) [file 10434_2019_7316_MOESM2_ESM.docx]

**Supplement 2. Content of the questionnaire**

| \| \|  \| \| --- \| \| \| --- \| --- \| |
| --- | --- | --- |
| *** Required Information** |

| \| \| **TIGER-study survey on the surgical treatment and extent of lymphadenectomy of esophageal cancer.   This web-based questionnaire is designed to get insight in the standpoint of the individual Surgeon in the worldwide controversy on the surgical approach and the extent of lymphadenectomy in the treatment of esophageal cancer.** \| \| --- \| \|  \| \| \| --- \| --- \| --- \| \| \| page 1 \| \| --- \| \| \| \| \| **1. Age responder. Fill out your age in years the text box below, use a number only (e.g. 42).** (Enter a value between 1 and 100) \| \| --- \| \| ____________________________________________________________________  ____________________________________________________________________ \| \| \| --- \| --- \| --- \| \| \|  \| \| \| \| \|  \| **2.** \| **What is your country of residence?** \| \| --- \| --- \| --- \| \| \| --- \| --- \| --- \| --- \| \|  \| \| \| --- \| --- \| --- \| --- \| --- \| --- \| \| \|  \| \| \| \| **3. Type of Medical Center you work in:** (Select one option) \| \| --- \| \| \|  \|  \| Local hospital \|  \| \| --- \| --- \| --- \| --- \| \|  \|  \| Secondary referral center \|  \| \|  \|  \| Tertiary referral center \|  \| \|  \|  \| Private clinic \|  \| \|  \|  \| Other (Please specify)  __________ \|  \| \| \| \| --- \| --- \| --- \| --- \| --- \| --- \| --- \| --- \| --- \| --- \| --- \| --- \| --- \| --- \| --- \| --- \| --- \| --- \| --- \| --- \| --- \| --- \| --- \| \| \|  \| \| \| **Surgical Experience:** \| \| --- \| \| \| **4. Fill out the number of years that you gained experience in** \| \| --- \| \| \| (a) \| General Surgery (since end of residency) \| \| --- \| --- \| \| \|  \| \| \| (b) \| Esophageal Surgery (since end of fellowship) \| \| --- \| --- \| \| \|  \| \| \| \|  \| \| \| **Case volumes:** \| \| --- \| \| \| **5. Fill out the number that corresponds to** \| \| --- \| \| \| (a) \| The number of esophagectomies in your Medical Center anually? \| \| --- \| --- \| \| \|  \| \| \| (b) \| The number of surgeons performing esophagectomies in your Medical Center anually? \| \| --- \| --- \| \| \|  \| \| \| (c) \| Your personal annual case volume? \| \| --- \| --- \| \| \|  \| \| \| | |
| --- | --- | --- | --- | --- | --- | --- | --- | --- | --- | --- | --- | --- | --- | --- | --- | --- | --- | --- | --- | --- | --- | --- | --- | --- | --- | --- | --- | --- | --- | --- | --- | --- | --- | --- | --- | --- | --- | --- | --- | --- | --- | --- | --- | --- | --- | --- | --- | --- | --- | --- | --- | --- | --- | --- | --- | --- | --- | --- | --- | --- | --- | --- | --- | --- | --- | --- | --- | --- | --- | --- | --- | --- | --- |
| \| \| page 2 \| \| --- \| \| \| --- \| --- \| \| \| \| *** 6. What definitions do you use for Mapping the Esophageal lymph nodes?** (Select one option) \| \| --- \| \| \| \|  \| \| --- \| \|  \| JSED 9th edition of esophageal cancer staging \|  \| Go to Page No. 3 \| \| --- \| --- \| --- \| --- \| --- \| --- \| \| \|  \| \| --- \| \|  \| AJCC 6th edition esophageal cancer staging \|  \| Go to Page No. 4 \| \|  \|  \| Other (Please specify)  __________ \| Go to Page No. 3 \|  \| \| \|  \| \| --- \| \| \| \| \|  \| \| \| \| --- \| --- \| --- \| --- \| --- \| --- \| --- \| --- \| --- \| --- \| --- \| --- \| --- \| --- \| --- \| --- \| --- \| --- \| --- \| --- \| --- \| --- \| --- \| --- \| --- \| --- \| \| \|  \| |  |

| \| \| page 3 \| \| --- \| \| \| --- \| --- \| \| \| \| \|  \| \|  \| \| --- \| \|  \| \|  \| \| --- \| --- \| --- \| --- \| --- \| \| \| --- \| --- \| --- \| --- \| --- \| --- \| \| *** 7. Which Lymph nodes do you resect in PROXIMAL Esophageal Squamous Cell Carcinoma? Please check all the boxes that match lymph nodes you remove according to the JSED 9th edition of esophageal cancer staging.  If you resect any lymph nodes that are not represented by a number on this map or in the legenda, please use the field other to specify. Also, if you differentiate between left and right nodes within a certain lymph node group, please use the field other to specify.** \| \| \|  \|  \| 100 Superficial cervical (R,L) \|  \| \| --- \| --- \| --- \| --- \| \|  \|  \| 101 Cervical paraesophageal (R,L) \|  \| \|  \|  \| 102 Deep Cervical (R,L) \|  \| \|  \|  \| 103 Peripharyngeal (R,L) \|  \| \|  \|  \| 104 Supraclavicular \|  \| \|  \|  \| 105 Upper Thoracic Paraesophageal \|  \| \|  \|  \| 108 Middle Thoracic Paraesophageal \|  \| \|  \|  \| 110 Lower Thoracic Paraesophageal \|  \| \|  \|  \| 106 Recurrent Nerve Right \|  \| \|  \|  \| 106 Recurrent Nerve Left \|  \| \|  \|  \| 106 Pretracheal \|  \| \|  \|  \| 106 Tracheobronchial Left \|  \| \|  \|  \| 107 Bifurcational \|  \| \|  \|  \| 109 Main Stem Bronchus (R,L) \|  \| \|  \|  \| 111 Supradiaphragmatic \|  \| \|  \|  \| 112 Posterior Mediastinal \|  \| \|  \|  \| 113 Ligamentum Arteriosum \|  \| \|  \|  \| 114 Anterior Mediastinal \|  \| \|  \|  \| 1,2 Cardiac (R, L) \|  \| \|  \|  \| 3 Lesser Curvature \|  \| \|  \|  \| 4 Greater Curvature \|  \| \|  \|  \| 7 Left Gastric Artery \|  \| \|  \|  \| 8 Common Hepatic Artery \|  \| \|  \|  \| 11 Splenic Artery \|  \| \|  \|  \| 9 Celiac Artery \|  \| \|  \|  \| Other (Please specify)  ______________ \| \| \| \| \| --- \| --- \| --- \| --- \| --- \| --- \| --- \| --- \| --- \| --- \| --- \| --- \| --- \| --- \| --- \| --- \| --- \| --- \| --- \| --- \| --- \| --- \| --- \| --- \| --- \| --- \| --- \| --- \| --- \| --- \| --- \| --- \| --- \| --- \| --- \| --- \| --- \| --- \| --- \| --- \| --- \| --- \| --- \| --- \| --- \| --- \| --- \| --- \| --- \| --- \| --- \| --- \| --- \| --- \| --- \| --- \| --- \| --- \| --- \| --- \| --- \| --- \| --- \| --- \| --- \| --- \| --- \| --- \| --- \| --- \| --- \| --- \| --- \| --- \| --- \| --- \| --- \| --- \| --- \| --- \| --- \| --- \| --- \| --- \| --- \| --- \| --- \| --- \| --- \| --- \| --- \| --- \| --- \| --- \| --- \| --- \| --- \| --- \| --- \| --- \| --- \| --- \| --- \| --- \| --- \| --- \| --- \| --- \| --- \| --- \| --- \| --- \| --- \| \| \|  \| \| \| \| \|  \| \|  \| \| --- \| \|  \| \|  \| \| --- \| --- \| --- \| --- \| --- \| \| \| --- \| --- \| --- \| --- \| --- \| --- \| \| *** 8. Which Lymph nodes do you resect in MID Esophageal Squamous Cell Carcinoma? Please check all the boxes that match lymph nodes you remove according to the JSED 9th edition of esophageal cancer staging.  If you resect any lymph nodes that are not represented by a number on this map or in the legenda, please use the field other to specify. Also, if you differentiate between left and right nodes within a certain lymph node group, please use the field other to specify.** \| \| \|  \|  \| 100 Superficial cervical (R,L) \|  \| \| --- \| --- \| --- \| --- \| \|  \|  \| 101 Cervical paraesophageal (R,L) \|  \| \|  \|  \| 102 Deep Cervical (R,L) \|  \| \|  \|  \| 103 Peripharyngeal (R,L) \|  \| \|  \|  \| 104 Supraclavicular \|  \| \|  \|  \| 105 Upper Thoracic Paraesophageal \|  \| \|  \|  \| 108 Middle Thoracic Paraesophageal \|  \| \|  \|  \| 110 Lower Thoracic Paraesophageal \|  \| \|  \|  \| 106 Recurrent Nerve Right \|  \| \|  \|  \| 106 Recurrent Nerve Left \|  \| \|  \|  \| 106 Pretracheal \|  \| \|  \|  \| 106 Tracheobronchial Left \|  \| \|  \|  \| 107 Bifurcational \|  \| \|  \|  \| 109 Main Stem Bronchus (R,L) \|  \| \|  \|  \| 111 Supradiaphragmatic \|  \| \|  \|  \| 112 Posterior Mediastinal \|  \| \|  \|  \| 113 Ligamentum Arteriosum \|  \| \|  \|  \| 114 Anterior Mediastinal \|  \| \|  \|  \| 1,2 Cardiac (R, L) \|  \| \|  \|  \| 3 Lesser Curvature \|  \| \|  \|  \| 4 Greater Curvature \|  \| \|  \|  \| 7 Left Gastric Artery \|  \| \|  \|  \| 8 Common Hepatic Artery \|  \| \|  \|  \| 11 Splenic Artery \|  \| \|  \|  \| 9 Celiac Artery \|  \| \|  \|  \| Other (Please specify)  ______________ \| \| \| \| \| --- \| --- \| --- \| --- \| --- \| --- \| --- \| --- \| --- \| --- \| --- \| --- \| --- \| --- \| --- \| --- \| --- \| --- \| --- \| --- \| --- \| --- \| --- \| --- \| --- \| --- \| --- \| --- \| --- \| --- \| --- \| --- \| --- \| --- \| --- \| --- \| --- \| --- \| --- \| --- \| --- \| --- \| --- \| --- \| --- \| --- \| --- \| --- \| --- \| --- \| --- \| --- \| --- \| --- \| --- \| --- \| --- \| --- \| --- \| --- \| --- \| --- \| --- \| --- \| --- \| --- \| --- \| --- \| --- \| --- \| --- \| --- \| --- \| --- \| --- \| --- \| --- \| --- \| --- \| --- \| --- \| --- \| --- \| --- \| --- \| --- \| --- \| --- \| --- \| --- \| --- \| --- \| --- \| --- \| --- \| --- \| --- \| --- \| --- \| --- \| --- \| --- \| --- \| --- \| --- \| --- \| --- \| --- \| --- \| --- \| --- \| --- \| --- \| \| \|  \| \| \| \| \|  \| \|  \| \| --- \| \|  \| \|  \| \| --- \| --- \| --- \| --- \| --- \| \| \| --- \| --- \| --- \| --- \| --- \| --- \| \| *** 9. Which Lymph nodes do you resect in DISTAL Esophageal Squamous Cell Carcinoma? Please check all the boxes that match lymph nodes you remove according to the JSED 9th edition of esophageal cancer staging.  If you resect any lymph nodes that are not represented by a number on this map or in the legenda, please use the field other to specify. Also, if you differentiate between left and right nodes within a certain lymph node group, please use the field other to specify.** \| \| \|  \|  \| 100 Superficial cervical (R,L) \|  \| \| --- \| --- \| --- \| --- \| \|  \|  \| 101 Cervical paraesophageal (R,L) \|  \| \|  \|  \| 102 Deep Cervical (R,L) \|  \| \|  \|  \| 103 Peripharyngeal (R,L) \|  \| \|  \|  \| 104 Supraclavicular \|  \| \|  \|  \| 105 Upper Thoracic Paraesophageal \|  \| \|  \|  \| 108 Middle Thoracic Paraesophageal \|  \| \|  \|  \| 110 Lower Thoracic Paraesophageal \|  \| \|  \|  \| 106 Recurrent Nerve Right \|  \| \|  \|  \| 106 Recurrent Nerve Left \|  \| \|  \|  \| 106 Pretracheal \|  \| \|  \|  \| 106 Tracheobronchial Left \|  \| \|  \|  \| 107 Bifurcational \|  \| \|  \|  \| 109 Main Stem Bronchus (R,L) \|  \| \|  \|  \| 111 Supradiaphragmatic \|  \| \|  \|  \| 112 Posterior Mediastinal \|  \| \|  \|  \| 113 Ligamentum Arteriosum \|  \| \|  \|  \| 114 Anterior Mediastinal \|  \| \|  \|  \| 1,2 Cardiac (R, L) \|  \| \|  \|  \| 3 Lesser Curvature \|  \| \|  \|  \| 4 Greater Curvature \|  \| \|  \|  \| 7 Left Gastric Artery \|  \| \|  \|  \| 8 Common Hepatic Artery \|  \| \|  \|  \| 11 Splenic Artery \|  \| \|  \|  \| 9 Celiac Artery \|  \| \|  \|  \| Other (Please specify)  ______________ \| \| \| \| \| --- \| --- \| --- \| --- \| --- \| --- \| --- \| --- \| --- \| --- \| --- \| --- \| --- \| --- \| --- \| --- \| --- \| --- \| --- \| --- \| --- \| --- \| --- \| --- \| --- \| --- \| --- \| --- \| --- \| --- \| --- \| --- \| --- \| --- \| --- \| --- \| --- \| --- \| --- \| --- \| --- \| --- \| --- \| --- \| --- \| --- \| --- \| --- \| --- \| --- \| --- \| --- \| --- \| --- \| --- \| --- \| --- \| --- \| --- \| --- \| --- \| --- \| --- \| --- \| --- \| --- \| --- \| --- \| --- \| --- \| --- \| --- \| --- \| --- \| --- \| --- \| --- \| --- \| --- \| --- \| --- \| --- \| --- \| --- \| --- \| --- \| --- \| --- \| --- \| --- \| --- \| --- \| --- \| --- \| --- \| --- \| --- \| --- \| --- \| --- \| --- \| --- \| --- \| --- \| --- \| --- \| --- \| --- \| --- \| --- \| --- \| --- \| --- \| \| \|  \| \| \| \| \|  \| \|  \| \| --- \| \|  \| \|  \| \| --- \| --- \| --- \| --- \| --- \| \| \| --- \| --- \| --- \| --- \| --- \| --- \| \| *** 10. Which Lymph nodes do you resect in SIEWERT type I adenocarcinoma of the esophagus? Please check all the boxes that match lymph nodes you remove according to the JSED 9th edition of esophageal cancer staging.  If you resect any lymph nodes that are not represented by a number on this map or in the legenda, please use the field other to specify. Also, if you differentiate between left and right nodes within a certain lymph node group, please use the field other to specify.** \| \| \|  \|  \| 100 Superficial cervical (R,L) \|  \| \| --- \| --- \| --- \| --- \| \|  \|  \| 101 Cervical paraesophageal (R,L) \|  \| \|  \|  \| 102 Deep Cervical (R,L) \|  \| \|  \|  \| 103 Peripharyngeal (R,L) \|  \| \|  \|  \| 104 Supraclavicular \|  \| \|  \|  \| 105 Upper Thoracic Paraesophageal \|  \| \|  \|  \| 108 Middle Thoracic Paraesophageal \|  \| \|  \|  \| 110 Lower Thoracic Paraesophageal \|  \| \|  \|  \| 106 Recurrent Nerve Right \|  \| \|  \|  \| 106 Recurrent Nerve Left \|  \| \|  \|  \| 106 Pretracheal \|  \| \|  \|  \| 106 Tracheobronchial Left \|  \| \|  \|  \| 107 Bifurcational \|  \| \|  \|  \| 109 Main Stem Bronchus (R,L) \|  \| \|  \|  \| 111 Supradiaphragmatic \|  \| \|  \|  \| 112 Posterior Mediastinal \|  \| \|  \|  \| 113 Ligamentum Arteriosum \|  \| \|  \|  \| 114 Anterior Mediastinal \|  \| \|  \|  \| 1,2 Cardiac (R, L) \|  \| \|  \|  \| 3 Lesser Curvature \|  \| \|  \|  \| 4 Greater Curvature \|  \| \|  \|  \| 7 Left Gastric Artery \|  \| \|  \|  \| 8 Common Hepatic Artery \|  \| \|  \|  \| 11 Splenic Artery \|  \| \|  \|  \| 9 Celiac Artery \|  \| \|  \|  \| Other (Please specify)  ______________ \| \| \| \| \| --- \| --- \| --- \| --- \| --- \| --- \| --- \| --- \| --- \| --- \| --- \| --- \| --- \| --- \| --- \| --- \| --- \| --- \| --- \| --- \| --- \| --- \| --- \| --- \| --- \| --- \| --- \| --- \| --- \| --- \| --- \| --- \| --- \| --- \| --- \| --- \| --- \| --- \| --- \| --- \| --- \| --- \| --- \| --- \| --- \| --- \| --- \| --- \| --- \| --- \| --- \| --- \| --- \| --- \| --- \| --- \| --- \| --- \| --- \| --- \| --- \| --- \| --- \| --- \| --- \| --- \| --- \| --- \| --- \| --- \| --- \| --- \| --- \| --- \| --- \| --- \| --- \| --- \| --- \| --- \| --- \| --- \| --- \| --- \| --- \| --- \| --- \| --- \| --- \| --- \| --- \| --- \| --- \| --- \| --- \| --- \| --- \| --- \| --- \| --- \| --- \| --- \| --- \| --- \| --- \| --- \| --- \| --- \| --- \| --- \| --- \| --- \| --- \| \| \|  \| \| \| \| \|  \| \|  \| \| --- \| \|  \| \|  \| \| --- \| --- \| --- \| --- \| --- \| \| \| --- \| --- \| --- \| --- \| --- \| --- \| \| *** 11. Which Lymph nodes do you resect in SIEWERT type II adenocarcinoma of the esophagus? Please check all the boxes that match lymph nodes you remove according to the JSED 9th edition of esophageal cancer staging.  If you resect any lymph nodes that are not represented by a number on this map or in the legenda, please use the field other to specify. Also, if you differentiate between left and right nodes within a certain lymph node group, please use the field other to specify.** \| \| \|  \|  \| 100 Superficial cervical (R,L) \|  \| \| --- \| --- \| --- \| --- \| \|  \|  \| 101 Cervical paraesophageal (R,L) \|  \| \|  \|  \| 102 Deep Cervical (R,L) \|  \| \|  \|  \| 103 Peripharyngeal (R,L) \|  \| \|  \|  \| 104 Supraclavicular \|  \| \|  \|  \| 105 Upper Thoracic Paraesophageal \|  \| \|  \|  \| 108 Middle Thoracic Paraesophageal \|  \| \|  \|  \| 110 Lower Thoracic Paraesophageal \|  \| \|  \|  \| 106 Recurrent Nerve Right \|  \| \|  \|  \| 106 Recurrent Nerve Left \|  \| \|  \|  \| 106 Pretracheal \|  \| \|  \|  \| 106 Tracheobronchial Left \|  \| \|  \|  \| 107 Bifurcational \|  \| \|  \|  \| 109 Main Stem Bronchus (R,L) \|  \| \|  \|  \| 111 Supradiaphragmatic \|  \| \|  \|  \| 112 Posterior Mediastinal \|  \| \|  \|  \| 113 Ligamentum Arteriosum \|  \| \|  \|  \| 114 Anterior Mediastinal \|  \| \|  \|  \| 1,2 Cardiac (R, L) \|  \| \|  \|  \| 3 Lesser Curvature \|  \| \|  \|  \| 4 Greater Curvature \|  \| \|  \|  \| 7 Left Gastric Artery \|  \| \|  \|  \| 8 Common Hepatic Artery \|  \| \|  \|  \| 11 Splenic Artery \|  \| \|  \|  \| 9 Celiac Artery \|  \| \|  \|  \| Other (Please specify)  ______________ \| \| \| \| \| --- \| --- \| --- \| --- \| --- \| --- \| --- \| --- \| --- \| --- \| --- \| --- \| --- \| --- \| --- \| --- \| --- \| --- \| --- \| --- \| --- \| --- \| --- \| --- \| --- \| --- \| --- \| --- \| --- \| --- \| --- \| --- \| --- \| --- \| --- \| --- \| --- \| --- \| --- \| --- \| --- \| --- \| --- \| --- \| --- \| --- \| --- \| --- \| --- \| --- \| --- \| --- \| --- \| --- \| --- \| --- \| --- \| --- \| --- \| --- \| --- \| --- \| --- \| --- \| --- \| --- \| --- \| --- \| --- \| --- \| --- \| --- \| --- \| --- \| --- \| --- \| --- \| --- \| --- \| --- \| --- \| --- \| --- \| --- \| --- \| --- \| --- \| --- \| --- \| --- \| --- \| --- \| --- \| --- \| --- \| --- \| --- \| --- \| --- \| --- \| --- \| --- \| --- \| --- \| --- \| --- \| --- \| --- \| --- \| --- \| --- \| --- \| --- \| \| \|  \| \| \| \| \|  \| \|  \| \| --- \| \|  \| \|  \| \| --- \| --- \| --- \| --- \| --- \| \| \| --- \| --- \| --- \| --- \| --- \| --- \| \| *** 12. Which Lymph nodes do you resect in SIEWERT type III adenocarcinoma of the esophagus? Please check all the boxes that match lymph nodes you remove according to the JSED 9th edition of esophageal cancer staging.  If you resect any lymph nodes that are not represented by a number on this map or in the legenda, please use the field other to specify. Also, if you differentiate between left and right nodes within a certain lymph node group, please use the field other to specify. Additionally, use the field other to specify whether you perform a D1 or D2 lymphadenectomy.** \| \| \|  \|  \| 100 Superficial cervical (R,L) \|  \| \| --- \| --- \| --- \| --- \| \|  \|  \| 101 Cervical paraesophageal (R,L) \|  \| \|  \|  \| 102 Deep Cervical (R,L) \|  \| \|  \|  \| 103 Peripharyngeal (R,L) \|  \| \|  \|  \| 104 Supraclavicular \|  \| \|  \|  \| 105 Upper Thoracic Paraesophageal \|  \| \|  \|  \| 108 Middle Thoracic Paraesophageal \|  \| \|  \|  \| 110 Lower Thoracic Paraesophageal \|  \| \|  \|  \| 106 Recurrent Nerve Right \|  \| \|  \|  \| 106 Recurrent Nerve Left \|  \| \|  \|  \| 106 Pretracheal \|  \| \|  \|  \| 106 Tracheobronchial Left \|  \| \|  \|  \| 107 Bifurcational \|  \| \|  \|  \| 109 Main Stem Bronchus (R,L) \|  \| \|  \|  \| 111 Supradiaphragmatic \|  \| \|  \|  \| 112 Posterior Mediastinal \|  \| \|  \|  \| 113 Ligamentum Arteriosum \|  \| \|  \|  \| 114 Anterior Mediastinal \|  \| \|  \|  \| 1,2 Cardiac (R, L) \|  \| \|  \|  \| 3 Lesser Curvature \|  \| \|  \|  \| 4 Greater Curvature \|  \| \|  \|  \| 7 Left Gastric Artery \|  \| \|  \|  \| 8 Common Hepatic Artery \|  \| \|  \|  \| 11 Splenic Artery \|  \| \|  \|  \| 9 Celiac Artery \|  \| \|  \|  \| Other (Please specify, and additionally differentiate between a D1 and D2 lymphadenectomy)  ______________ \| \| \| \| \| Go to Page No. 5 \| \| --- \| \|  \| \| \| \| --- \| --- \| --- \| --- \| --- \| --- \| --- \| --- \| --- \| --- \| --- \| --- \| --- \| --- \| --- \| --- \| --- \| --- \| --- \| --- \| --- \| --- \| --- \| --- \| --- \| --- \| --- \| --- \| --- \| --- \| --- \| --- \| --- \| --- \| --- \| --- \| --- \| --- \| --- \| --- \| --- \| --- \| --- \| --- \| --- \| --- \| --- \| --- \| --- \| --- \| --- \| --- \| --- \| --- \| --- \| --- \| --- \| --- \| --- \| --- \| --- \| --- \| --- \| --- \| --- \| --- \| --- \| --- \| --- \| --- \| --- \| --- \| --- \| --- \| --- \| --- \| --- \| --- \| --- \| --- \| --- \| --- \| --- \| --- \| --- \| --- \| --- \| --- \| --- \| --- \| --- \| --- \| --- \| --- \| --- \| --- \| --- \| --- \| --- \| --- \| --- \| --- \| --- \| --- \| --- \| --- \| --- \| --- \| --- \| --- \| --- \| --- \| --- \| --- \| --- \| --- \| \| \|  \| |
| --- | --- | --- | --- | --- | --- | --- | --- | --- | --- | --- | --- | --- | --- | --- | --- | --- | --- | --- | --- | --- | --- | --- | --- | --- | --- | --- | --- | --- | --- | --- | --- | --- | --- | --- | --- | --- | --- | --- | --- | --- | --- | --- | --- | --- | --- | --- | --- | --- | --- | --- | --- | --- | --- | --- | --- | --- | --- | --- | --- | --- | --- | --- | --- | --- | --- | --- | --- | --- | --- | --- | --- | --- | --- | --- | --- | --- | --- | --- | --- | --- | --- | --- | --- | --- | --- | --- | --- | --- | --- | --- | --- | --- | --- | --- | --- | --- | --- | --- | --- | --- | --- | --- | --- | --- | --- | --- | --- | --- | --- | --- | --- | --- | --- | --- | --- | --- | --- | --- | --- | --- | --- | --- | --- | --- | --- | --- | --- | --- | --- | --- | --- | --- | --- | --- | --- | --- | --- | --- | --- | --- | --- | --- | --- | --- | --- | --- | --- | --- | --- | --- | --- | --- | --- | --- | --- | --- | --- | --- | --- | --- | --- | --- | --- | --- | --- | --- | --- | --- | --- | --- | --- | --- | --- | --- | --- | --- | --- | --- | --- | --- | --- | --- | --- | --- | --- | --- | --- | --- | --- | --- | --- | --- | --- | --- | --- | --- | --- | --- | --- | --- | --- | --- | --- | --- | --- | --- | --- | --- | --- | --- | --- | --- | --- | --- | --- | --- | --- | --- | --- | --- | --- | --- | --- | --- | --- | --- | --- | --- | --- | --- | --- | --- | --- | --- | --- | --- | --- | --- | --- | --- | --- | --- | --- | --- | --- | --- | --- | --- | --- | --- | --- | --- | --- | --- | --- | --- | --- | --- | --- | --- | --- | --- | --- | --- | --- | --- | --- | --- | --- | --- | --- | --- | --- | --- | --- | --- | --- | --- | --- | --- | --- | --- | --- | --- | --- | --- | --- | --- | --- | --- | --- | --- | --- | --- | --- | --- | --- | --- | --- | --- | --- | --- | --- | --- | --- | --- | --- | --- | --- | --- | --- | --- | --- | --- | --- | --- | --- | --- | --- | --- | --- | --- | --- | --- | --- | --- | --- | --- | --- | --- | --- | --- | --- | --- | --- | --- | --- | --- | --- | --- | --- | --- | --- | --- | --- | --- | --- | --- | --- | --- | --- | --- | --- | --- | --- | --- | --- | --- | --- | --- | --- | --- | --- | --- | --- | --- | --- | --- | --- | --- | --- | --- | --- | --- | --- | --- | --- | --- | --- | --- | --- | --- | --- | --- | --- | --- | --- | --- | --- | --- | --- | --- | --- | --- | --- | --- | --- | --- | --- | --- | --- | --- | --- | --- | --- | --- | --- | --- | --- | --- | --- | --- | --- | --- | --- | --- | --- | --- | --- | --- | --- | --- | --- | --- | --- | --- | --- | --- | --- | --- | --- | --- | --- | --- | --- | --- | --- | --- | --- | --- | --- | --- | --- | --- | --- | --- | --- | --- | --- | --- | --- | --- | --- | --- | --- | --- | --- | --- | --- | --- | --- | --- | --- | --- | --- | --- | --- | --- | --- | --- | --- | --- | --- | --- | --- | --- | --- | --- | --- | --- | --- | --- | --- | --- | --- | --- | --- | --- | --- | --- | --- | --- | --- | --- | --- | --- | --- | --- | --- | --- | --- | --- | --- | --- | --- | --- | --- | --- | --- | --- | --- | --- | --- | --- | --- | --- | --- | --- | --- | --- | --- | --- | --- | --- | --- | --- | --- | --- | --- | --- | --- | --- | --- | --- | --- | --- | --- | --- | --- | --- | --- | --- | --- | --- | --- | --- | --- | --- | --- | --- | --- | --- | --- | --- | --- | --- | --- | --- | --- | --- | --- | --- | --- | --- | --- | --- | --- | --- | --- | --- | --- | --- | --- | --- | --- | --- | --- | --- | --- | --- | --- | --- | --- | --- | --- | --- | --- | --- | --- | --- | --- | --- | --- | --- | --- | --- | --- | --- | --- | --- | --- | --- | --- | --- | --- | --- | --- | --- | --- | --- | --- | --- | --- | --- | --- | --- | --- | --- | --- | --- | --- | --- | --- | --- | --- | --- | --- | --- | --- | --- | --- | --- | --- | --- | --- | --- | --- | --- | --- | --- | --- | --- | --- | --- | --- | --- | --- | --- | --- | --- | --- | --- | --- | --- | --- | --- | --- | --- | --- | --- | --- | --- | --- | --- | --- | --- | --- | --- | --- | --- | --- | --- | --- | --- | --- | --- | --- | --- | --- | --- | --- | --- | --- | --- | --- | --- | --- | --- | --- | --- | --- | --- | --- | --- | --- |

| \|  \| \| --- \| \| \| page 4 \| \| --- \| \| \| \| \| \|  \| \|  \| \| --- \| \|  \| \|  \| \| --- \| --- \| --- \| --- \| --- \| \| \| --- \| --- \| --- \| --- \| --- \| --- \| \| *** 13. Which Lymph nodes do you resect in PROXIMAL Esophageal Squamous Cell Carcinoma? Please check all the boxes that match lymph nodes you remove according to the AJCC 6th edition of esophageal cancer staging.  If you resect any lymph nodes that are not represented by a number on this map or in the legenda, please use the field other to specify. Also, if you differentiate between left and right nodes within a certain lymph node group, please use the field other to specify.** \| \| \|  \|  \| 1 Supraclavicular nodes \|  \| \| --- \| --- \| --- \| --- \| \|  \|  \| 2R Right upper paratrachel nodes \|  \| \|  \|  \| 2L Left upper paratracheal nodes \|  \| \|  \|  \| 3P Posterior Mediastinal nodes \|  \| \|  \|  \| 4R Right lower paratracheal nodes \|  \| \|  \|  \| 4L Left lower paratracheal nodes \|  \| \|  \|  \| 5 Aortopulmonary nodes \|  \| \|  \|  \| 6 Anterior mediastinal nodes \|  \| \|  \|  \| 7 Subcarinal nodes \|  \| \|  \|  \| 8M Middle paraesophageal nodes \|  \| \|  \|  \| 8L Lower paraesophageal nodes \|  \| \|  \|  \| 9 Pulmonary ligament nodes \|  \| \|  \|  \| 10R Right tracheobronchial nodes \|  \| \|  \|  \| 10L Left tracheobronchial nodes \|  \| \|  \|  \| 15 Diaphragmatic nodes \|  \| \|  \|  \| 16 Paracardial nodes \|  \| \|  \|  \| 17 Left gastric nodes \|  \| \|  \|  \| 18 Common hepatic nodes \|  \| \|  \|  \| 19 Splenic nodes \|  \| \|  \|  \| 20 Celiac nodes \|  \| \|  \|  \| Other (Please specify)  ______________ \| \| \| \| \| --- \| --- \| --- \| --- \| --- \| --- \| --- \| --- \| --- \| --- \| --- \| --- \| --- \| --- \| --- \| --- \| --- \| --- \| --- \| --- \| --- \| --- \| --- \| --- \| --- \| --- \| --- \| --- \| --- \| --- \| --- \| --- \| --- \| --- \| --- \| --- \| --- \| --- \| --- \| --- \| --- \| --- \| --- \| --- \| --- \| --- \| --- \| --- \| --- \| --- \| --- \| --- \| --- \| --- \| --- \| --- \| --- \| --- \| --- \| --- \| --- \| --- \| --- \| --- \| --- \| --- \| --- \| --- \| --- \| --- \| --- \| --- \| --- \| --- \| --- \| --- \| --- \| --- \| --- \| --- \| --- \| --- \| --- \| --- \| --- \| --- \| --- \| --- \| --- \| --- \| --- \| --- \| --- \| \| \|  \| \| \| \| \|  \| \|  \| \| --- \| \|  \| \|  \| \| --- \| --- \| --- \| --- \| --- \| \| \| --- \| --- \| --- \| --- \| --- \| --- \| \| *** 14. Which Lymph nodes do you resect in MID Esophageal Squamous Cell Carcinoma? Please check all the boxes that match lymph nodes you remove according to the AJCC 6th edition of esophageal cancer staging.  If you resect any lymph nodes that are not represented by a number on this map or in the legenda, please use the field other to specify. Also, if you differentiate between left and right nodes within a certain lymph node group, please use the field other to specify.** \| \| \|  \|  \| 1 Supraclavicular nodes \|  \| \| --- \| --- \| --- \| --- \| \|  \|  \| 2R Right upper paratrachel nodes \|  \| \|  \|  \| 2L Left upper paratracheal nodes \|  \| \|  \|  \| 3P Posterior Mediastinal nodes \|  \| \|  \|  \| 4R Right lower paratracheal nodes \|  \| \|  \|  \| 4L Left lower paratracheal nodes \|  \| \|  \|  \| 5 Aortopulmonary nodes \|  \| \|  \|  \| 6 Anterior mediastinal nodes \|  \| \|  \|  \| 7 Subcarinal nodes \|  \| \|  \|  \| 8M Middle paraesophageal nodes \|  \| \|  \|  \| 8L Lower paraesophageal nodes \|  \| \|  \|  \| 9 Pulmonary ligament nodes \|  \| \|  \|  \| 10R Right tracheobronchial nodes \|  \| \|  \|  \| 10L Left tracheobronchial nodes \|  \| \|  \|  \| 15 Diaphragmatic nodes \|  \| \|  \|  \| 16 Paracardial nodes \|  \| \|  \|  \| 17 Left gastric nodes \|  \| \|  \|  \| 18 Common hepatic nodes \|  \| \|  \|  \| 19 Splenic nodes \|  \| \|  \|  \| 20 Celiac nodes \|  \| \|  \|  \| Other (Please specify)  ______________ \| \| \| \| \| --- \| --- \| --- \| --- \| --- \| --- \| --- \| --- \| --- \| --- \| --- \| --- \| --- \| --- \| --- \| --- \| --- \| --- \| --- \| --- \| --- \| --- \| --- \| --- \| --- \| --- \| --- \| --- \| --- \| --- \| --- \| --- \| --- \| --- \| --- \| --- \| --- \| --- \| --- \| --- \| --- \| --- \| --- \| --- \| --- \| --- \| --- \| --- \| --- \| --- \| --- \| --- \| --- \| --- \| --- \| --- \| --- \| --- \| --- \| --- \| --- \| --- \| --- \| --- \| --- \| --- \| --- \| --- \| --- \| --- \| --- \| --- \| --- \| --- \| --- \| --- \| --- \| --- \| --- \| --- \| --- \| --- \| --- \| --- \| --- \| --- \| --- \| --- \| --- \| --- \| --- \| --- \| --- \| \| \|  \| \| \| \| \|  \| \|  \| \| --- \| \|  \| \|  \| \| --- \| --- \| --- \| --- \| --- \| \| \| --- \| --- \| --- \| --- \| --- \| --- \| \| *** 15. Which Lymph nodes do you resect in DISTAL Esophageal Squamous Cell Carcinoma? Please check all the boxes that match lymph nodes you remove according to the AJCC 6th edition of esophageal cancer staging.  If you resect any lymph nodes that are not represented by a number on this map or in the legenda, please use the field other to specify. Also, if you differentiate between left and right nodes within a certain lymph node group, please use the field other to specify.** \| \| \|  \|  \| 1 Supraclavicular nodes \|  \| \| --- \| --- \| --- \| --- \| \|  \|  \| 2R Right upper paratrachel nodes \|  \| \|  \|  \| 2L Left upper paratracheal nodes \|  \| \|  \|  \| 3P Posterior Mediastinal nodes \|  \| \|  \|  \| 4R Right lower paratracheal nodes \|  \| \|  \|  \| 4L Left lower paratracheal nodes \|  \| \|  \|  \| 5 Aortopulmonary nodes \|  \| \|  \|  \| 6 Anterior mediastinal nodes \|  \| \|  \|  \| 7 Subcarinal nodes \|  \| \|  \|  \| 8M Middle paraesophageal nodes \|  \| \|  \|  \| 8L Lower paraesophageal nodes \|  \| \|  \|  \| 9 Pulmonary ligament nodes \|  \| \|  \|  \| 10R Right tracheobronchial nodes \|  \| \|  \|  \| 10L Left tracheobronchial nodes \|  \| \|  \|  \| 15 Diaphragmatic nodes \|  \| \|  \|  \| 16 Paracardial nodes \|  \| \|  \|  \| 17 Left gastric nodes \|  \| \|  \|  \| 18 Common hepatic nodes \|  \| \|  \|  \| 19 Splenic nodes \|  \| \|  \|  \| 20 Celiac nodes \|  \| \|  \|  \| Other (Please specify)  ______________ \| \| \| \| \| --- \| --- \| --- \| --- \| --- \| --- \| --- \| --- \| --- \| --- \| --- \| --- \| --- \| --- \| --- \| --- \| --- \| --- \| --- \| --- \| --- \| --- \| --- \| --- \| --- \| --- \| --- \| --- \| --- \| --- \| --- \| --- \| --- \| --- \| --- \| --- \| --- \| --- \| --- \| --- \| --- \| --- \| --- \| --- \| --- \| --- \| --- \| --- \| --- \| --- \| --- \| --- \| --- \| --- \| --- \| --- \| --- \| --- \| --- \| --- \| --- \| --- \| --- \| --- \| --- \| --- \| --- \| --- \| --- \| --- \| --- \| --- \| --- \| --- \| --- \| --- \| --- \| --- \| --- \| --- \| --- \| --- \| --- \| --- \| --- \| --- \| --- \| --- \| --- \| --- \| --- \| --- \| --- \| \| \|  \| \| \| \| \|  \| \|  \| \| --- \| \|  \| \|  \| \| --- \| --- \| --- \| --- \| --- \| \| \| --- \| --- \| --- \| --- \| --- \| --- \| \| *** 16. Which Lymph nodes do you resect in SIEWERT type I adenocarcinoma of the esophagus? Please check all the boxes that match lymph nodes you remove according to the AJCC 6th edition of esophageal cancer staging.  If you resect any lymph nodes that are not represented by a number on this map or in the legenda, please use the field other to specify. Also, if you differentiate between left and right nodes within a certain lymph node group, please use the field other to specify.** \| \| \|  \|  \| 1 Supraclavicular nodes \|  \| \| --- \| --- \| --- \| --- \| \|  \|  \| 2R Right upper paratrachel nodes \|  \| \|  \|  \| 2L Left upper paratracheal nodes \|  \| \|  \|  \| 3P Posterior Mediastinal nodes \|  \| \|  \|  \| 4R Right lower paratracheal nodes \|  \| \|  \|  \| 4L Left lower paratracheal nodes \|  \| \|  \|  \| 5 Aortopulmonary nodes \|  \| \|  \|  \| 6 Anterior mediastinal nodes \|  \| \|  \|  \| 7 Subcarinal nodes \|  \| \|  \|  \| 8M Middle paraesophageal nodes \|  \| \|  \|  \| 8L Lower paraesophageal nodes \|  \| \|  \|  \| 9 Pulmonary ligament nodes \|  \| \|  \|  \| 10R Right tracheobronchial nodes \|  \| \|  \|  \| 10L Left tracheobronchial nodes \|  \| \|  \|  \| 15 Diaphragmatic nodes \|  \| \|  \|  \| 16 Paracardial nodes \|  \| \|  \|  \| 17 Left gastric nodes \|  \| \|  \|  \| 18 Common hepatic nodes \|  \| \|  \|  \| 19 Splenic nodes \|  \| \|  \|  \| 20 Celiac nodes \|  \| \|  \|  \| Other (Please specify)  ______________ \| \| \| \| \| --- \| --- \| --- \| --- \| --- \| --- \| --- \| --- \| --- \| --- \| --- \| --- \| --- \| --- \| --- \| --- \| --- \| --- \| --- \| --- \| --- \| --- \| --- \| --- \| --- \| --- \| --- \| --- \| --- \| --- \| --- \| --- \| --- \| --- \| --- \| --- \| --- \| --- \| --- \| --- \| --- \| --- \| --- \| --- \| --- \| --- \| --- \| --- \| --- \| --- \| --- \| --- \| --- \| --- \| --- \| --- \| --- \| --- \| --- \| --- \| --- \| --- \| --- \| --- \| --- \| --- \| --- \| --- \| --- \| --- \| --- \| --- \| --- \| --- \| --- \| --- \| --- \| --- \| --- \| --- \| --- \| --- \| --- \| --- \| --- \| --- \| --- \| --- \| --- \| --- \| --- \| --- \| --- \| \| \|  \| \| \| \| \|  \| \|  \| \| --- \| \|  \| \|  \| \| --- \| --- \| --- \| --- \| --- \| \| \| --- \| --- \| --- \| --- \| --- \| --- \| \| *** 17. Which Lymph nodes do you resect in SIEWERT type II adenocarcinoma of the esophagus? Please check all the boxes that match lymph nodes you remove according to the AJCC 6th edition of esophageal cancer staging.  If you resect any lymph nodes that are not represented by a number on this map or in the legenda, please use the field other to specify. Also, if you differentiate between left and right nodes within a certain lymph node group, please use the field other to specify.** \| \| \|  \|  \| 1 Supraclavicular nodes \|  \| \| --- \| --- \| --- \| --- \| \|  \|  \| 2R Right upper paratrachel nodes \|  \| \|  \|  \| 2L Left upper paratracheal nodes \|  \| \|  \|  \| 3P Posterior Mediastinal nodes \|  \| \|  \|  \| 4R Right lower paratracheal nodes \|  \| \|  \|  \| 4L Left lower paratracheal nodes \|  \| \|  \|  \| 5 Aortopulmonary nodes \|  \| \|  \|  \| 6 Anterior mediastinal nodes \|  \| \|  \|  \| 7 Subcarinal nodes \|  \| \|  \|  \| 8M Middle paraesophageal nodes \|  \| \|  \|  \| 8L Lower paraesophageal nodes \|  \| \|  \|  \| 9 Pulmonary ligament nodes \|  \| \|  \|  \| 10R Right tracheobronchial nodes \|  \| \|  \|  \| 10L Left tracheobronchial nodes \|  \| \|  \|  \| 15 Diaphragmatic nodes \|  \| \|  \|  \| 16 Paracardial nodes \|  \| \|  \|  \| 17 Left gastric nodes \|  \| \|  \|  \| 18 Common hepatic nodes \|  \| \|  \|  \| 19 Splenic nodes \|  \| \|  \|  \| 20 Celiac nodes \|  \| \|  \|  \| Other (Please specify)  ______________ \| \| \| \| \| --- \| --- \| --- \| --- \| --- \| --- \| --- \| --- \| --- \| --- \| --- \| --- \| --- \| --- \| --- \| --- \| --- \| --- \| --- \| --- \| --- \| --- \| --- \| --- \| --- \| --- \| --- \| --- \| --- \| --- \| --- \| --- \| --- \| --- \| --- \| --- \| --- \| --- \| --- \| --- \| --- \| --- \| --- \| --- \| --- \| --- \| --- \| --- \| --- \| --- \| --- \| --- \| --- \| --- \| --- \| --- \| --- \| --- \| --- \| --- \| --- \| --- \| --- \| --- \| --- \| --- \| --- \| --- \| --- \| --- \| --- \| --- \| --- \| --- \| --- \| --- \| --- \| --- \| --- \| --- \| --- \| --- \| --- \| --- \| --- \| --- \| --- \| --- \| --- \| --- \| --- \| --- \| --- \| \| \|  \| \| \| \| \|  \| \|  \| \| --- \| \|  \| \|  \| \| --- \| --- \| --- \| --- \| --- \| \| \| --- \| --- \| --- \| --- \| --- \| --- \| \| *** 18. Which Lymph nodes do you resect in SIEWERT type III adenocarcinoma of the esophagus? Please check all the boxes that match lymph nodes you remove according to the AJCC 6th edition of esophageal cancer staging.  If you resect any lymph nodes that are not represented by a number on this map or in the legenda, please use the field other to specify. Also, if you differentiate between left and right nodes within a certain lymph node group, please use the field other to specify. Additionally, use the field other to specify whether you perform a D1 or D2 lymphadenectomy.** \| \| \|  \|  \| 1 Supraclavicular nodes \|  \| \| --- \| --- \| --- \| --- \| \|  \|  \| 2R Right upper paratrachel nodes \|  \| \|  \|  \| 2L Left upper paratracheal nodes \|  \| \|  \|  \| 3P Posterior Mediastinal nodes \|  \| \|  \|  \| 4R Right lower paratracheal nodes \|  \| \|  \|  \| 4L Left lower paratracheal nodes \|  \| \|  \|  \| 5 Aortopulmonary nodes \|  \| \|  \|  \| 6 Anterior mediastinal nodes \|  \| \|  \|  \| 7 Subcarinal nodes \|  \| \|  \|  \| 8M Middle paraesophageal nodes \|  \| \|  \|  \| 8L Lower paraesophageal nodes \|  \| \|  \|  \| 9 Pulmonary ligament nodes \|  \| \|  \|  \| 10R Right tracheobronchial nodes \|  \| \|  \|  \| 10L Left tracheobronchial nodes \|  \| \|  \|  \| 15 Diaphragmatic nodes \|  \| \|  \|  \| 16 Paracardial nodes \|  \| \|  \|  \| 17 Left gastric nodes \|  \| \|  \|  \| 18 Common hepatic nodes \|  \| \|  \|  \| 19 Splenic nodes \|  \| \|  \|  \| 20 Celiac nodes \|  \| \|  \|  \| Other (Please specify, and additionally differentiate between a D1 and D2 lymphadenectomy)  ______________ \| \| \| \| \| --- \| --- \| --- \| --- \| --- \| --- \| --- \| --- \| --- \| --- \| --- \| --- \| --- \| --- \| --- \| --- \| --- \| --- \| --- \| --- \| --- \| --- \| --- \| --- \| --- \| --- \| --- \| --- \| --- \| --- \| --- \| --- \| --- \| --- \| --- \| --- \| --- \| --- \| --- \| --- \| --- \| --- \| --- \| --- \| --- \| --- \| --- \| --- \| --- \| --- \| --- \| --- \| --- \| --- \| --- \| --- \| --- \| --- \| --- \| --- \| --- \| --- \| --- \| --- \| --- \| --- \| --- \| --- \| --- \| --- \| --- \| --- \| --- \| --- \| --- \| --- \| --- \| --- \| --- \| --- \| --- \| --- \| --- \| --- \| --- \| --- \| --- \| --- \| --- \| --- \| --- \| --- \| --- \| \| \|  \| |
| --- | --- | --- | --- | --- | --- | --- | --- | --- | --- | --- | --- | --- | --- | --- | --- | --- | --- | --- | --- | --- | --- | --- | --- | --- | --- | --- | --- | --- | --- | --- | --- | --- | --- | --- | --- | --- | --- | --- | --- | --- | --- | --- | --- | --- | --- | --- | --- | --- | --- | --- | --- | --- | --- | --- | --- | --- | --- | --- | --- | --- | --- | --- | --- | --- | --- | --- | --- | --- | --- | --- | --- | --- | --- | --- | --- | --- | --- | --- | --- | --- | --- | --- | --- | --- | --- | --- | --- | --- | --- | --- | --- | --- | --- | --- | --- | --- | --- | --- | --- | --- | --- | --- | --- | --- | --- | --- | --- | --- | --- | --- | --- | --- | --- | --- | --- | --- | --- | --- | --- | --- | --- | --- | --- | --- | --- | --- | --- | --- | --- | --- | --- | --- | --- | --- | --- | --- | --- | --- | --- | --- | --- | --- | --- | --- | --- | --- | --- | --- | --- | --- | --- | --- | --- | --- | --- | --- | --- | --- | --- | --- | --- | --- | --- | --- | --- | --- | --- | --- | --- | --- | --- | --- | --- | --- | --- | --- | --- | --- | --- | --- | --- | --- | --- | --- | --- | --- | --- | --- | --- | --- | --- | --- | --- | --- | --- | --- | --- | --- | --- | --- | --- | --- | --- | --- | --- | --- | --- | --- | --- | --- | --- | --- | --- | --- | --- | --- | --- | --- | --- | --- | --- | --- | --- | --- | --- | --- | --- | --- | --- | --- | --- | --- | --- | --- | --- | --- | --- | --- | --- | --- | --- | --- | --- | --- | --- | --- | --- | --- | --- | --- | --- | --- | --- | --- | --- | --- | --- | --- | --- | --- | --- | --- | --- | --- | --- | --- | --- | --- | --- | --- | --- | --- | --- | --- | --- | --- | --- | --- | --- | --- | --- | --- | --- | --- | --- | --- | --- | --- | --- | --- | --- | --- | --- | --- | --- | --- | --- | --- | --- | --- | --- | --- | --- | --- | --- | --- | --- | --- | --- | --- | --- | --- | --- | --- | --- | --- | --- | --- | --- | --- | --- | --- | --- | --- | --- | --- | --- | --- | --- | --- | --- | --- | --- | --- | --- | --- | --- | --- | --- | --- | --- | --- | --- | --- | --- | --- | --- | --- | --- | --- | --- | --- | --- | --- | --- | --- | --- | --- | --- | --- | --- | --- | --- | --- | --- | --- | --- | --- | --- | --- | --- | --- | --- | --- | --- | --- | --- | --- | --- | --- | --- | --- | --- | --- | --- | --- | --- | --- | --- | --- | --- | --- | --- | --- | --- | --- | --- | --- | --- | --- | --- | --- | --- | --- | --- | --- | --- | --- | --- | --- | --- | --- | --- | --- | --- | --- | --- | --- | --- | --- | --- | --- | --- | --- | --- | --- | --- | --- | --- | --- | --- | --- | --- | --- | --- | --- | --- | --- | --- | --- | --- | --- | --- | --- | --- | --- | --- | --- | --- | --- | --- | --- | --- | --- | --- | --- | --- | --- | --- | --- | --- | --- | --- | --- | --- | --- | --- | --- | --- | --- | --- | --- | --- | --- | --- | --- | --- | --- | --- | --- | --- | --- | --- | --- | --- | --- | --- | --- | --- | --- | --- | --- | --- | --- | --- | --- | --- | --- | --- | --- | --- | --- | --- | --- | --- | --- | --- | --- | --- | --- | --- | --- | --- | --- | --- | --- | --- | --- | --- | --- | --- | --- | --- | --- | --- | --- | --- | --- | --- | --- | --- | --- | --- | --- | --- | --- | --- | --- | --- | --- | --- | --- | --- | --- | --- | --- | --- | --- | --- | --- | --- | --- | --- | --- | --- | --- | --- | --- | --- | --- | --- | --- | --- | --- | --- | --- | --- | --- | --- | --- | --- | --- | --- |

| \|  \| \| --- \| \| \| page 5 \| \| --- \| \| \| \| \| **19. Please provide the percentages (to a total maximum of 100%) of histologic types of Esophageal Carcinoma in your Medical Center.** \| \| --- \| \| \| \| Adenocarcinoma \| \| --- \| \|  \| \| --- \| --- \| --- \| \| \| Squamous Cell Carcinoma \| \| --- \| \|  \| \| \| Other types of Esophageal Carcinoma \| \| --- \| \|  \| \|  \|  \| \| \| \| --- \| --- \| --- \| --- \| --- \| --- \| --- \| --- \| --- \| --- \| --- \| --- \| --- \| --- \| \| \|  \| \| \| \| \|  \| **20.** \| **Is a Sentinel Lymph node procedure used in your Medical Center to determine the extent of lymphadenectomy? (Select one option)** \| \| --- \| --- \| --- \| \| \| --- \| --- \| --- \| --- \| \| \|  \|  \| No Sentinel Lymph node procedure is used. \|  \| \| --- \| --- \| --- \| --- \| \|  \|  \| Yes, a SLN procedure is used. Please specify the technique (imaging, including radiocolloid or dye)  __________ \|  \| \| \| \| --- \| --- \| --- \| --- \| --- \| --- \| --- \| --- \| --- \| --- \| --- \| --- \| --- \| --- \| \| \|  \| \| \| \| *** 21. Do patients with Esophageal Carcinoma receive neo-adjuvant therapy in your Medical Center?** (Select one option) \| \| --- \| \| \|  \|  \| Yes. \|  \| Go to Page No. 6 \| \| --- \| --- \| --- \| --- \| --- \| \|  \|  \| No. \|  \| Go to Page No. 7 \| \| \| If Did Not Answer Then Go to Page No. 6 \| \| --- \| \| \| \| \|  \| \| \| \| --- \| --- \| --- \| --- \| --- \| --- \| --- \| --- \| --- \| --- \| --- \| --- \| --- \| --- \| --- \| --- \| --- \| --- \| --- \| \| |
| --- | --- | --- | --- | --- | --- | --- | --- | --- | --- | --- | --- | --- | --- | --- | --- | --- | --- | --- | --- | --- | --- | --- | --- | --- | --- | --- | --- | --- | --- | --- | --- | --- | --- | --- | --- | --- | --- | --- | --- | --- | --- | --- | --- | --- | --- | --- | --- | --- | --- | --- | --- | --- | --- | --- | --- |

| \| \|  \| \| --- \| \| \| --- \| --- \| \| \| page 6 \| \| --- \| \| \| \| \| **22. Specify the type of neo-adjuvant therapy used in your Medical Center for Proximal Esophageal Squamous Cell Carcinoma.  If no option is applicable, use the text box and specify in detail what type of neo-adjuvant therapy is used. EXAMPLES: - Name the chemotherapeutic agent(s) and its scheme - Radiotherapy ..... Gy in ..... fractions - Name the chemotherapeutic agent(s) and its scheme combined with Radiotherapy ..... Gy in ..... fractions - Name the immunotherapeutic agent(s) and its scheme** (Select one option) \| \| --- \| \| \|  \|  \| CROSS-scheme (5 cycles of Paclitaxel and Carboplatin with concurrent radiotherapy - 23 fractions of 1.8 Gy) \|  \| \| --- \| --- \| --- \| --- \| \|  \|  \| MAGIC- scheme (3 cycles of Epirubicin, Cisplatin and Capecitabine / 5-FU) \|  \| \|  \|  \| Other (specify)  __________ \|  \| \| \| \| --- \| --- \| --- \| --- \| --- \| --- \| --- \| --- \| --- \| --- \| --- \| --- \| --- \| --- \| --- \| \| \|  \| \| \| \| **23. Specify the type of neo-adjuvant therapy used in your Medical Center for Mid Esophageal Squamous Cell Carcinoma. If no option is applicable, use the text box and specify in detail what type of neo-adjuvant therapy is used. EXAMPLES: - Name the chemotherapeutic agent(s) and its scheme - Radiotherapy ..... Gy in ..... fractions - Name the chemotherapeutic agent(s) and its scheme combined with Radiotherapy ..... Gy in ..... fractions - Name the immunotherapeutic agent(s) and its scheme** (Select one option) \| \| --- \| \| \|  \|  \| CROSS-scheme (5 cycles of Paclitaxel and Carboplatin with concurrent radiotherapy - 23 fractions of 1.8 Gy) \|  \| \| --- \| --- \| --- \| --- \| \|  \|  \| MAGIC- scheme (3 cycles of Epirubicin, Cisplatin and Capecitabine / 5-FU) \|  \| \|  \|  \| Other (specify)  __________ \|  \| \| \| \| --- \| --- \| --- \| --- \| --- \| --- \| --- \| --- \| --- \| --- \| --- \| --- \| --- \| --- \| --- \| \| \|  \| \| \| \| **24. Specify the type of neo-adjuvant therapy used in your Medical Center for Distal Esophageal Squamous Cell Carcinoma. If no option is applicable, use the text box and specify in detail what type of neo-adjuvant therapy is used. EXAMPLES: - Name the chemotherapeutic agent(s) and its scheme - Radiotherapy ..... Gy in ..... fractions - Name the chemotherapeutic agent(s) and its scheme combined with Radiotherapy ..... Gy in ..... fractions - Name the immunotherapeutic agent(s) and its scheme** (Select one option) \| \| --- \| \| \|  \|  \| CROSS-scheme (5 cycles of Paclitaxel and Carboplatin with concurrent radiotherapy - 23 fractions of 1.8 Gy) \|  \| \| --- \| --- \| --- \| --- \| \|  \|  \| MAGIC- scheme (3 cycles of Epirubicin, Cisplatin and Capecitabine / 5-FU) \|  \| \|  \|  \| Other (specify)  __________ \|  \| \| \| \| --- \| --- \| --- \| --- \| --- \| --- \| --- \| --- \| --- \| --- \| --- \| --- \| --- \| --- \| --- \| \| \|  \| \| \| \| **25. Specify the type of neo-adjuvant therapy used in your Medical Center for Siewert type I adenocarcinoma. If no option is applicable, use the text box and specify in detail what type of neo-adjuvant therapy is used. EXAMPLES: - Name the chemotherapeutic agent(s) and its scheme - Radiotherapy ..... Gy in ..... fractions - Name the chemotherapeutic agent(s) and its scheme combined with Radiotherapy ..... Gy in ..... fractions - Name the immunotherapeutic agent(s) and its scheme** (Select one option) \| \| --- \| \| \|  \|  \| CROSS-scheme (5 cycles of Paclitaxel and Carboplatin with concurrent radiotherapy - 23 fractions of 1.8 Gy) \|  \| \| --- \| --- \| --- \| --- \| \|  \|  \| MAGIC- scheme (3 cycles of Epirubicin, Cisplatin and Capecitabine / 5-FU) \|  \| \|  \|  \| Other (specify)  __________ \|  \| \| \| \| --- \| --- \| --- \| --- \| --- \| --- \| --- \| --- \| --- \| --- \| --- \| --- \| --- \| --- \| --- \| \| \|  \| \| \| \| **26. Specify the type of neo-adjuvant therapy used in your Medical Center for Siewert type II adenocarcinoma. If no option is applicable, use the text box and specify in detail what type of neo-adjuvant therapy is used. EXAMPLES: - Name the chemotherapeutic agent(s) and its scheme - Radiotherapy ..... Gy in ..... fractions - Name the chemotherapeutic agent(s) and its scheme combined with Radiotherapy ..... Gy in ..... fractions - Name the immunotherapeutic agent(s) and its scheme** (Select one option) \| \| --- \| \| \|  \|  \| CROSS-scheme (5 cycles of Paclitaxel and Carboplatin with concurrent radiotherapy - 23 fractions of 1.8 Gy) \|  \| \| --- \| --- \| --- \| --- \| \|  \|  \| MAGIC- scheme (3 cycles of Epirubicin, Cisplatin and Capecitabine / 5-FU) \|  \| \|  \|  \| Other (specify)  __________ \|  \| \| \| \| --- \| --- \| --- \| --- \| --- \| --- \| --- \| --- \| --- \| --- \| --- \| --- \| --- \| --- \| --- \| \| \|  \| \| \| \| **27. Specify the type of neo-adjuvant therapy used in your Medical Center for Siewert type III adenocarcinoma. If no option is applicable, use the text box and specify in detail what type of neo-adjuvant therapy is used. EXAMPLES: - Name the chemotherapeutic agent(s) and its scheme - Radiotherapy ..... Gy in ..... fractions - Name the chemotherapeutic agent(s) and its scheme combined with Radiotherapy ..... Gy in ..... fractions - Name the immunotherapeutic agent(s) and its scheme** (Select one option) \| \| --- \| \| \|  \|  \| CROSS-scheme (5 cycles of Paclitaxel and Carboplatin with concurrent radiotherapy - 23 fractions of 1.8 Gy) \|  \| \| --- \| --- \| --- \| --- \| \|  \|  \| MAGIC- scheme (3 cycles of Epirubicin, Cisplatin and Capecitabine / 5-FU) \|  \| \|  \|  \| Other (specify)  __________ \|  \| \| \| \| --- \| --- \| --- \| --- \| --- \| --- \| --- \| --- \| --- \| --- \| --- \| --- \| --- \| --- \| --- \| \| \|  \| \| \| \| \|  \| **28.** \| **Does neo-adjuvant treatment influence the esophagectomy? (e.g. does neo-adjuvant treatment make the esophagectomy more difficult by its influence on the tissue, more time-comsuming etc.) (Select one option)** \| \| --- \| --- \| --- \| \| \| --- \| --- \| --- \| --- \| \| \|  \|  \| No \|  \| \| --- \| --- \| --- \| --- \| \|  \|  \| Yes (please use the text box that appears to specify the influence on the esophagectomy)  __________ \|  \| \| \| \| --- \| --- \| --- \| --- \| --- \| --- \| --- \| --- \| --- \| --- \| --- \| --- \| --- \| --- \| \| \|  \| \| \| \| **29. Does neo-adjuvant therapy influence the extent of lymphadenectomy in your Medical Center?** (Select one option) \| \| --- \| \| \|  \|  \| Yes, a less extensive lymphadenectomy is performed. \|  \| \| --- \| --- \| --- \| --- \| \|  \|  \| Yes, a more extensive lymphadenectomy is perfomed. \|  \| \|  \|  \| No. \|  \| \| \| \| --- \| --- \| --- \| --- \| --- \| --- \| --- \| --- \| --- \| --- \| --- \| --- \| --- \| --- \| --- \| \| \|  \| |
| --- | --- | --- | --- | --- | --- | --- | --- | --- | --- | --- | --- | --- | --- | --- | --- | --- | --- | --- | --- | --- | --- | --- | --- | --- | --- | --- | --- | --- | --- | --- | --- | --- | --- | --- | --- | --- | --- | --- | --- | --- | --- | --- | --- | --- | --- | --- | --- | --- | --- | --- | --- | --- | --- | --- | --- | --- | --- | --- | --- | --- | --- | --- | --- | --- | --- | --- | --- | --- | --- | --- | --- | --- | --- | --- | --- | --- | --- | --- | --- | --- | --- | --- | --- | --- | --- | --- | --- | --- | --- | --- | --- | --- | --- | --- | --- | --- | --- | --- | --- | --- | --- | --- | --- | --- | --- | --- | --- | --- | --- | --- | --- | --- | --- | --- | --- | --- | --- | --- | --- | --- | --- | --- | --- | --- | --- | --- | --- | --- | --- | --- | --- | --- | --- | --- | --- | --- | --- | --- | --- |

| \| \|  \| \| --- \| \| \| --- \| --- \| \| \| page 7 \| \| --- \| \| \| \| \| **30. What type of surgery do you preferably perform?** (Select one option) \| \| --- \| \| \|  \|  \| Open \|  \| \| --- \| --- \| --- \| --- \| \|  \|  \| Minimally Invasive \|  \| \| \| \| --- \| --- \| --- \| --- \| --- \| --- \| --- \| --- \| --- \| --- \| --- \| \| \| \| **What is your preferred surgical strategy in** \| \| --- \| \| \| \| **31. Choose the type of sugery for each type of esophageal carcinoma; multiple options per type of carcinoma can be checked** \| \| --- \| \| \| \| \| \| \| \| \| \| \| \| \| \| \| \| \| \| \| \| \| \| \| \| \| \| \| \| \| \| \| \| \| \| \| \| \| \| \| \| \| \| \| \| \| \| \| \| \| \| \| \| \| --- \| --- \| --- \| --- \| --- \| --- \| --- \| --- \| --- \| --- \| --- \| --- \| --- \| --- \| --- \| --- \| --- \| --- \| --- \| --- \| --- \| --- \| --- \| --- \| --- \| --- \| --- \| --- \| --- \| --- \| --- \| --- \| --- \| --- \| --- \| --- \| --- \| --- \| --- \| --- \| --- \| --- \| --- \| --- \| --- \| --- \| --- \| --- \| --- \| --- \| --- \| --- \| \|  \| **Gastrectomy** \| **Transhiatal** \| **Transthoracic, intrathoracic anastomosis** \| **Transthoracic, cervical anastomosis** \| **Other** \|  \|  \|  \|  \|  \|  \|  \|  \|  \|  \|  \|  \|  \|  \|  \|  \|  \|  \|  \|  \|  \|  \|  \|  \|  \|  \|  \|  \|  \|  \|  \|  \|  \|  \|  \|  \|  \|  \|  \|  \|  \|  \|  \|  \|  \| \| \|  \| (a) \| Proximal SCC \| \| --- \| --- \| --- \| \| \|  \|  \| \| --- \| --- \| \| \|  \|  \| \| --- \| --- \| \| \|  \|  \| \| --- \| --- \| \| \|  \|  \| \| --- \| --- \| \| \|  \|  \| \| --- \| --- \| \|  \|  \|  \|  \|  \|  \|  \|  \|  \|  \|  \|  \|  \|  \|  \|  \|  \|  \|  \|  \|  \|  \|  \|  \|  \|  \|  \|  \|  \|  \|  \|  \|  \|  \|  \|  \|  \|  \|  \|  \|  \|  \|  \|  \|  \| \| \|  \| (b) \| Mid SCC \| \| --- \| --- \| --- \| \| \|  \|  \| \| --- \| --- \| \| \|  \|  \| \| --- \| --- \| \| \|  \|  \| \| --- \| --- \| \| \|  \|  \| \| --- \| --- \| \| \|  \|  \| \| --- \| --- \| \|  \|  \|  \|  \|  \|  \|  \|  \|  \|  \|  \|  \|  \|  \|  \|  \|  \|  \|  \|  \|  \|  \|  \|  \|  \|  \|  \|  \|  \|  \|  \|  \|  \|  \|  \|  \|  \|  \|  \|  \|  \|  \|  \|  \|  \| \| \|  \| (c) \| Distal SCC \| \| --- \| --- \| --- \| \| \|  \|  \| \| --- \| --- \| \| \|  \|  \| \| --- \| --- \| \| \|  \|  \| \| --- \| --- \| \| \|  \|  \| \| --- \| --- \| \| \|  \|  \| \| --- \| --- \| \|  \|  \|  \|  \|  \|  \|  \|  \|  \|  \|  \|  \|  \|  \|  \|  \|  \|  \|  \|  \|  \|  \|  \|  \|  \|  \|  \|  \|  \|  \|  \|  \|  \|  \|  \|  \|  \|  \|  \|  \|  \|  \|  \|  \|  \| \| \|  \| (d) \| Adeno Siewert I \| \| --- \| --- \| --- \| \| \|  \|  \| \| --- \| --- \| \| \|  \|  \| \| --- \| --- \| \| \|  \|  \| \| --- \| --- \| \| \|  \|  \| \| --- \| --- \| \| \|  \|  \| \| --- \| --- \| \|  \|  \|  \|  \|  \|  \|  \|  \|  \|  \|  \|  \|  \|  \|  \|  \|  \|  \|  \|  \|  \|  \|  \|  \|  \|  \|  \|  \|  \|  \|  \|  \|  \|  \|  \|  \|  \|  \|  \|  \|  \|  \|  \|  \|  \| \| \|  \| (e) \| Adeno Siewert II \| \| --- \| --- \| --- \| \| \|  \|  \| \| --- \| --- \| \| \|  \|  \| \| --- \| --- \| \| \|  \|  \| \| --- \| --- \| \| \|  \|  \| \| --- \| --- \| \| \|  \|  \| \| --- \| --- \| \|  \|  \|  \|  \|  \|  \|  \|  \|  \|  \|  \|  \|  \|  \|  \|  \|  \|  \|  \|  \|  \|  \|  \|  \|  \|  \|  \|  \|  \|  \|  \|  \|  \|  \|  \|  \|  \|  \|  \|  \|  \|  \|  \|  \|  \| \| \|  \| (f) \| Adeno Siewert III \| \| --- \| --- \| --- \| \| \|  \|  \| \| --- \| --- \| \| \|  \|  \| \| --- \| --- \| \| \|  \|  \| \| --- \| --- \| \| \|  \|  \| \| --- \| --- \| \| \|  \|  \| \| --- \| --- \| \|  \|  \|  \|  \|  \|  \|  \|  \|  \|  \|  \|  \|  \|  \|  \|  \|  \|  \|  \|  \|  \|  \|  \|  \|  \|  \|  \|  \|  \|  \|  \|  \|  \|  \|  \|  \|  \|  \|  \|  \|  \|  \|  \|  \|  \| \| \| \| **32. If you answered ''other'': please specify in the text box** \| \| --- \| \| \| \|  \| \| \| \| **33. How is the sprecimen resected?** (Select one option) \| \| --- \| \| \|  \|  \| En bloc \|  \| \| --- \| --- \| --- \| --- \| \|  \|  \| En bloc, lymph node stations marked by the surgeon with sutures or beads \|  \| \|  \|  \| With lymph node stations groups separated from the specimen by the surgeon and presented to the pathologist separately \|  \| \|  \|  \| Other (Please specify)  __________ \|  \| \| \| \| --- \| --- \| --- \| --- \| --- \| --- \| --- \| --- \| --- \| --- \| --- \| --- \| --- \| --- \| --- \| --- \| --- \| --- \| --- \| \| \| \| \| \|  \| **34.** \| **If you send lymph nodes separately to the pathologist, you separate the lymph nodes (Select one option) [ Answer this question only if answer to Q#33 is With lymph node stations groups separated from the specimen by the surgeon and presented to the pathologist separately ]** \| \| --- \| --- \| --- \| \| \| --- \| --- \| --- \| --- \| \| \|  \|  \| In vivo \|  \| \| --- \| --- \| --- \| --- \| \|  \|  \| Ex vivo \|  \| \|  \|  \| Partially in vivo and partially ex vivo \|  \| \|  \|  \| Other (Please specify)  __________ \|  \| \| \| \| --- \| --- \| --- \| --- \| --- \| --- \| --- \| --- \| --- \| --- \| --- \| --- \| --- \| --- \| --- \| --- \| --- \| --- \| --- \| --- \| --- \| --- \| \| \| \| \| \|  \| **35.** \| **If you send lymph node stations separately to the pathologist, lymph node station close to the primary tumor are (Select one option) [ Answer this question only if answer to Q#33 is With lymph node stations groups separated from the specimen by the surgeon and presented to the pathologist separately ]** \| \| --- \| --- \| --- \| \| \| --- \| --- \| --- \| --- \| \| \|  \|  \| Removed ex vivo \|  \| \| --- \| --- \| --- \| --- \| \|  \|  \| Removed in vivo \|  \| \|  \|  \| Marked with sutures or beads \|  \| \|  \|  \| Other (Please specify)  __________ \|  \| \| \| \| --- \| --- \| --- \| --- \| --- \| --- \| --- \| --- \| --- \| --- \| --- \| --- \| --- \| --- \| --- \| --- \| --- \| --- \| --- \| --- \| --- \| --- \| \| \| \| \| **36. If you have any remarks on this survey, please use the text box below.   Thank you for your participation!** \| \| --- \| \| \| --- \| --- \| \| \| \|  \| \| --- \| \| **All data collected in this survey will be handled confidentially. The data will be analyzed and published anonymously.** \| \| |
| --- | --- | --- | --- | --- | --- | --- | --- | --- | --- | --- | --- | --- | --- | --- | --- | --- | --- | --- | --- | --- | --- | --- | --- | --- | --- | --- | --- | --- | --- | --- | --- | --- | --- | --- | --- | --- | --- | --- | --- | --- | --- | --- | --- | --- | --- | --- | --- | --- | --- | --- | --- | --- | --- | --- | --- | --- | --- | --- | --- | --- | --- | --- | --- | --- | --- | --- | --- | --- | --- | --- | --- | --- | --- | --- | --- | --- | --- | --- | --- | --- | --- | --- | --- | --- | --- | --- | --- | --- | --- | --- | --- | --- | --- | --- | --- | --- | --- | --- | --- | --- | --- | --- | --- | --- | --- | --- | --- | --- | --- | --- | --- | --- | --- | --- | --- | --- | --- | --- | --- | --- | --- | --- | --- | --- | --- | --- | --- | --- | --- | --- | --- | --- | --- | --- | --- | --- | --- | --- | --- | --- | --- | --- | --- | --- | --- | --- | --- | --- | --- | --- | --- | --- | --- | --- | --- | --- | --- | --- | --- | --- | --- | --- | --- | --- | --- | --- | --- | --- | --- | --- | --- | --- | --- | --- | --- | --- | --- | --- | --- | --- | --- | --- | --- | --- | --- | --- | --- | --- | --- | --- | --- | --- | --- | --- | --- | --- | --- | --- | --- | --- | --- | --- | --- | --- | --- | --- | --- | --- | --- | --- | --- | --- | --- | --- | --- | --- | --- | --- | --- | --- | --- | --- | --- | --- | --- | --- | --- | --- | --- | --- | --- | --- | --- | --- | --- | --- | --- | --- | --- | --- | --- | --- | --- | --- | --- | --- | --- | --- | --- | --- | --- | --- | --- | --- | --- | --- | --- | --- | --- | --- | --- | --- | --- | --- | --- | --- | --- | --- | --- | --- | --- | --- | --- | --- | --- | --- | --- | --- | --- | --- | --- | --- | --- | --- | --- | --- | --- | --- | --- | --- | --- | --- | --- | --- | --- | --- | --- | --- | --- | --- | --- | --- | --- | --- | --- | --- | --- | --- | --- | --- | --- | --- | --- | --- | --- | --- | --- | --- | --- | --- | --- | --- | --- | --- | --- | --- | --- | --- | --- | --- | --- | --- | --- | --- | --- | --- | --- | --- | --- | --- | --- | --- | --- | --- | --- | --- | --- | --- | --- | --- | --- | --- | --- | --- | --- | --- | --- | --- | --- | --- | --- | --- | --- | --- | --- | --- | --- | --- | --- | --- | --- | --- | --- | --- | --- | --- | --- | --- | --- | --- | --- | --- | --- | --- | --- | --- | --- | --- | --- | --- | --- | --- | --- | --- | --- | --- | --- | --- | --- | --- | --- | --- | --- | --- | --- | --- | --- | --- | --- | --- | --- | --- | --- | --- | --- | --- | --- | --- | --- | --- | --- | --- | --- | --- | --- | --- | --- | --- | --- | --- | --- | --- | --- | --- | --- | --- | --- | --- | --- | --- | --- | --- | --- | --- | --- | --- | --- | --- | --- | --- | --- | --- | --- | --- | --- | --- | --- | --- | --- | --- | --- | --- | --- | --- | --- | --- | --- | --- | --- | --- | --- | --- | --- | --- | --- | --- | --- | --- | --- | --- | --- | --- | --- | --- | --- | --- | --- | --- | --- | --- | --- | --- | --- | --- | --- | --- | --- | --- | --- | --- | --- | --- | --- | --- | --- | --- | --- | --- | --- | --- | --- | --- | --- | --- | --- | --- | --- | --- | --- | --- | --- | --- | --- | --- | --- | --- | --- | --- | --- | --- | --- | --- | --- | --- | --- | --- | --- | --- | --- | --- | --- | --- | --- | --- | --- | --- | --- | --- | --- | --- | --- | --- | --- | --- | --- | --- | --- | --- | --- | --- | --- | --- | --- | --- | --- | --- | --- | --- | --- | --- | --- | --- | --- | --- | --- | --- | --- | --- | --- | --- | --- |
